# Disentangling task-selection failures from task-execution failures in task switching: an assessment of different paradigms

**DOI:** 10.1007/s00426-022-01708-5

**Published:** 2022-07-14

**Authors:** Luca Moretti, Iring Koch, Marco Steinhauser, Stefanie Schuch

**Affiliations:** 1 Institute of Psychology, RWTH Aachen University, Jaegerstrasse 17/19, 52066 Aachen, Germany; 2 Catholic University of Eichstätt-Ingolstadt, Eichstätt, Germany

## Abstract

Differentiating errors on the basis of the distinct cognitive mechanisms that may have generated them has provided neuropsychologists with useful diagnostic tools. For example, perseverative errors arising from the inability of the patient to set a new criterion for responding are considered one of the hallmarks of cognitive inflexibility. Similarly, in the task-switching paradigm it is possible to distinguish between task-confusion errors, produced by a failure in task selection, and response-confusion errors, arising when the correct task is selected, but the wrong response is given. Nonetheless, only a few studies so far have exploited the existence of different kinds of errors in multitasking situations to inform theories of cognitive flexibility. In the present study, we set out to use a variety of methodologies employed so far in the literature for disentangling errors due to task-selection failure from errors due to task-execution failure. In three experiments, we assessed the capacity of each method to produce error categories that can be mapped as clearly as possible to the cognitive mechanism(s) underlying them using multinomial processing tree modelling. Subsequently, the distinction between task- and response-confusion errors was used to test their differential impact on inhibitory mechanisms in task switching as measured by N-2 repetition costs. Our results are encouraging regarding the possibility of correctly detecting response- and task-selection failures, thus allowing us to assess their differential impact on N-2 repetition costs.

**Supplementary Information:**

The online version contains supplementary material available at 10.1007/s00426-022-01708-5.

## Introduction

While leaving his house, a man, immersed in his thoughts, starts walking the usual way to work, until he realises that it is Sunday and he was not planning on going to work at all, but rather on visiting his mother. He, therefore, changes direction, but when he gets to his mother’s house door, again thinking of something else, he tries to open it using his office key.

Although both mistakes made by the absent-minded man prevent him from reaching his goal (pay a visit to his mother), they greatly differ from one another when considering the underlying cognitive mechanisms producing them. From a classical cognitive control perspective, it is possible to distinguish between the activation of an action schema, automatically driven by sensory input (Gade & Koch, 2007a; Meiran & Kessler, 2008; Steinhauser & Gade, 2015; Yamaguchi & Proctor, 2011), and its implementation (Norman & Shallice, 1986; Stuss & Alexander, 2007; Stuss et al., 1995). As the man stands in front of his door he takes the most common and automatic route to work, and thus fails to set the appropriate goal for his actions (i.e. “visit mom”). He chooses the appropriate action (walking), but for the wrong task. When instead he attempts to open the door with the wrong key, he is correctly pursuing his goal (i.e. “open the door to visit mom”), but he is choosing the wrong action to implement it.

The present study is concerned with empirically disentangling errors arising from inappropriate task selection from those that are due to inappropriate task execution. After a brief review of the methodologies employed in previous behavioural studies for drawing this distinction, we will present three task-switching experiments implementing these different methodologies. This will allow for a direct comparison of different approaches for disentangling the two kinds of errors occurring in multi-tasking situations. In particular, multinomial processing tree models will be used to estimate how often task-selection failures and task-execution failures result in separate observable error categories, which have been used in previous literature as their empirical markers.

Furthermore, the (different) impact of these errors on task-switching performance is going to be assessed. Other than previous task-switching studies, which focused on the measure of task-switch costs (i.e., the performance difference between task switches and task repetitions), we will investigate the impact of (different) errors on an index of task inhibition, the N-2 repetition costs (i.e., the performance difference between task-switch sequences of type ABA vs. CBA), building up on a previous study from our lab (Moretti et al., 2021). Based on a task-strengthening account of task-switching performance (Steinhauser & Hübner, 2006), we predicted that N-2 repetition costs will be abolished following a task-selection failure in trial N-2, but not a task-execution failure.

## How to disentangle task-selection failures and task-execution failures?

The task-switching paradigm has been widely used to investigate cognitive flexibility in humans (Koch et al., 2018; Vandierendonck et al., 2010). In studies using this paradigm, participants are asked to rapidly switch among simple stimulus-judgment tasks such as indicating whether a digit is odd or even, or whether the same digit is greater or smaller than five.

Since the very beginning of research on cognitive flexibility, particular attention was devoted to different kinds of errors, distinguishing errors due to application of the no-longer relevant task set from other errors (Grant & Berg, 1948). Outside the neuropsychological tradition, however, this distinction has been less regarded (see Schuch et al., 2019, for a review of error processing in task switching). As outlined in the present paper, the occurrence of different error types can nonetheless be used also outside the clinical field for informing theories of cognitive flexibility. However, as outlined in the next section, unambiguously detecting the source of an error can be a non-trivial endeavour in task switching.

### The distinction between cognitive and observed error types

**Fig. 1:**
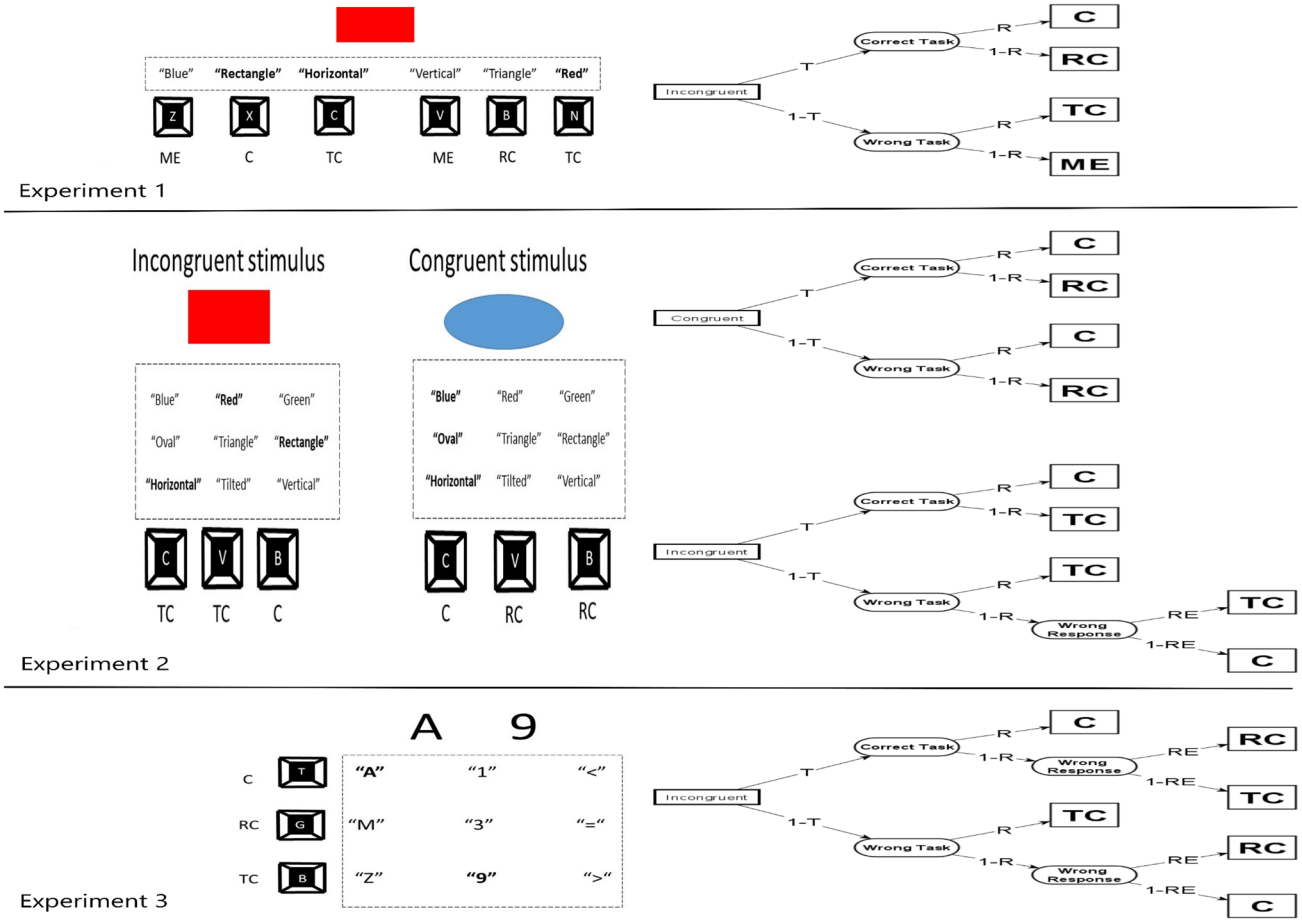
On the left: visual representation of the different methodologies used in Experiments 1, 2 and 3. At the top of each row, an example stimulus is depicted. In Experiments 1 and 2, the participant needs to perform the “Shape” task, in Experiment 3, the “Letter” task. Below each example stimulus, the words within the boxes represent all the possible abstract response categories present in a given experiment. Response categories marked in bold are those afforded by the example stimulus, of which only one represents the correct answer. The black boxes below (or next) one or more categories represent the keys used for indicating those categories. Finally, the letters below (or next) the response keys represent how that response would be classified in a given trial: C = Response classified as correct, RC = Response classified as response-confusion error, TC = Response classified as task-confusion error. ME = Response classified as a mixed error. On the right: Graphical representations of the multinomial processing trees. Rectangles on the left denote different trial types, each constituting different trees. From them different branches depart each terminating on a square on the right, representing observable response categories. Ovals in the middle represent unobservable cognitive events. Each unobservable event occurs with a probability expressed by the associated parameter on the link. Parameter T corresponds to the probability of selecting the required task in a given trial. Parameter R is the probability of choosing the correct response for the selected task. Parameter RE represents the probability that, having chosen the wrong response for the wrong task, accidentally leads to performing the correct response. As this is the result of a supposedly random process, this parameter was fixed to 0.5

One important issue when studying errors due to task-selection failure, and errors due to task-execution failure, is that these distinct cognitive processes do not always clearly map to observable behaviour in the laboratory. As such, for the present paper it is crucial to distinguish between these two levels (cognitive process vs. empirical phenomenon). When referring to the processing level, we will use the term *task-selection failure* to describe those situations in which the participant performs the currently irrelevant task, and *task-execution failure* to refer to those situations in which the correct task is selected, but the response is still wrongly executed. We will instead use the terms *task-confusion error* and *response-confusion* error to describe those observable events that we suppose to be (mostly) stemming from task-selection failures and task-execution failures, respectively. In what follows we provide a short review of the methodologies used in behavioural research to map the different error types to observable error categories (see Fig. 1). After this, we present three task-switching experiments using the reviewed methodologies to provide a direct comparison between them. Finally, the (different) impact of the task- and response-confusion errors on task-switching performance (focusing on N-2 repetition costs) will be assessed, allowing us to test critical predictions from the response-based strengthening account of task-switching performance (Steinhauser & Hübner, 2006), which posits that activated task-sets become strengthened upon their execution.

### Methodology I: univalent task-response mapping

Perhaps the most intuitive way to differentiate between errors due to task-selection failure versus task-execution failure is to use univalent task-response mappings (Desmet et al., 2011, 2012; Meiran & Daichman, 2005; see also Zheng et al., 2018), meaning that the response-set for each task is unique (see an upper panel of Fig. 1).

In a first study on the occurrence of task-selection failures, for example, participants had to indicate the spatial location of a stimulus presented in a 2 × 2 grid along the vertical (“top”/ “bottom”) or horizontal (“left”/” right”) dimension (Meiran & Daichman, 2005; see also: Meiran et al., 2001). To do so, they could press one of 4 arrow keys; the vertical task was mapped to two keys arranged vertically (to be pressed with the middle and index finger of the right hand), and the horizontal task was mapped to two separate keys, arranged horizontally (to be pressed with middle and index finger of the left hand). With this set-up, a response would be labelled as a task-confusion error if a key belonging to the response-set associated with the irrelevant task was pressed (e.g. if the relevant task was “vertical”, pressing either the “left” or “right” key). On the other hand, a response-confusion error would be present if the wrong key of the correct response-set was pressed (e.g. if the stimulus was “top”, but the participant pressed the “down” key). The results show that task-confusion errors were extremely rare (about 2%) when extensive preparation time was given (i.e. when the cue-stimulus interval [CSI] was long).

As Meiran and Daichman (2005) notice, despite the intuitive appeal of this methodology, it is possible that the effect of preparation time (reduction of task-confusion errors with long vs. short CSI) is not driven by task preparation, but rather by effector preparation. In other words, it is possible that when the task cue appears, the participants pre-activate the effectors they are going to use (e.g., middle and index finger of left hand). However, choosing one of these pre-activated effectors does not necessarily imply that the participants are actually attending to the relevant stimulus dimension, and hence, are performing the correct task. Therefore, many errors due to failures in task selection may go undetected with this methodology, and become classified as response-confusion errors.

### Methodology II: capitalizing on stimulus congruency

To circumvent the confound between effector selection and task selection, bivalent task-response mappings can be used (see the central panel of Fig. 1), where the same response set is used for responding to both tasks. In this case, however, it is less straightforward to distinguish between errors due to task-selection failure and errors due to task-execution failure.

Consider the example given in the previous paragraph. Having bivalent task-response mappings means that only two response keys are used for both tasks, e.g. one key indicates both “up” and “left”, and the other one “down” and “right” (Meiran & Daichman, 2005). In this context if a congruent stimulus is presented, meaning that the correct responses to the relevant and to the irrelevant stimulus dimensions are both mapped to the same key, it would be impossible to disentangle a correct response from the correct application of the irrelevant task. For example, if the stimulus is presented on the top-left part of the grid, and the task is to judge it on the vertical dimension, the correct response would be “up”. However, since in our example the same key is also used for indicating “left”, it is not clear whether the participant correctly indicated “up”, or rather they intended to indicate the currently irrelevant dimension of the stimulus, “left”. It follows that an error made on a congruent trial is most likely due to a task-execution failure, and can be classified as a response-confusion error. On the other hand, errors in incongruent trials, where the correct responses for the relevant and irrelevant tasks are spatially separated, can either be due to task-selection failure or task-execution failure.

Previous studies have often assumed that errors in incongruent trials would be due to task-selection failure much more frequently than errors in congruent trials (Ikeda & Hasegawa, 2012; Meiran & Daichman, 2005; Steinhauser & Hübner, 2006, 2008). To corroborate this assumption, multinomial-processing trees (MPT) models have been used to estimate the relative frequency of task-selection failures in congruent and incongruent trials (Meiran & Daichman, 2005; Steinhauser & Hübner, 2006; Steinhauser et al., 2017; Steinhauser & Steinhauser, 2019). MPT are a class of models that allow for estimating latent processes underlying observed frequency data (for a formal definition see: Riefer & Batchelder, 1988; Hu & Batchelder, 1994; for a review see: Erdfelder et al., 2009; we will explain MPT methodology in more detail in the General method section). Results from such MPT modelling show a rather large variability, with errors due to task-selection failures ranging between 19% (Meiran & Daichman, 2005) and 54.7% (Steinhauser & Steinhauser, 2019) in incongruent trials. Crucially, however, all of these studies found that task-selection failures were much more frequent (up to 10 times) in incongruent trials than in congruent trials, supporting the idea that comparing errors in incongruent versus congruent trials can be used as a proxy for disentangling errors due to task-selection failure and task-execution failure in task switching.

While MPT models provide several advantages, however, one limitation remains: other than the methodology of univalent response mappings, they do not allow for separating task-selection and task-execution failures on the level of single trials (for a possible solution to this issue, see Gluth & Meiran, 2019).

### Methodology III: using more response keys than levels of stimulus dimension

The most recently introduced method for disentangling task- and response-selection failures is that of using more response keys than responses afforded by the currently presented stimulus (see bottom panel of Fig. 1).

Typically, in this paradigm stimuli are composed of a target and a distractor presented spatially segregated (Steinhauser & Gade, 2015; Steinhauser et al., 2017; Steinhauser & Steinhauser, 2019). As only incongruent stimuli are used, in each trial one response key represents the correct response for the target (i.e. correct response), and another key is mapped to the correct response for the distractor (i.e. task-confusion error). Differently from the methodology presented in the previous section, however, a third key is present which is neither the correct response for the target, nor for the distractor (i.e. corresponding to a response-confusion error). This is made possible by the fact that each task has three response options; that is, each stimulus dimension varies on *three* levels, which are mapped to three separated keys.

For instance, in the aforementioned studies, a character-picture pair was presented in each trial, and participants were asked to either make a classification of the character while ignoring the picture, or the other way around. Crucially, the character dimension could vary on three levels, so that in each trial a character could be a letter, a numeral, or a symbol. In the same way, the pictures could be of animals, fruits, or vehicles. As such, one key was present for e.g. “letter” and “animal”, one for “numeral” and “fruit”, and one for “symbol” and “vehicle”. However, of these three keys, only two were mapped to the currently presented stimulus (e.g., letter and fruit).

Compared to the congruency-based methodology presented in the previous section, this methodology seems to provide a better way of disentangling the different error types, because here, failures in response selection can be measured more directly as the frequency of third-key responses (whereas no such direct measure exists in the method described in the previous section). In fact, task-selection failures with the three-response methodology have been estimated to be more prevalent among classified task-confusion errors (~ 50%; see Steinhauser et al., 2017; Steinhauser & Steinhauser, 2019) than with the congruency-based methodology presented in the previous paragraph (~ 20%; Meiran & Daichman, 2005; Steinhauser & Hübner, 2006).

Nonetheless, the limitation remains that MPT modelling does not allow for determining the error type on a trial-by-trial basis, but only allows for estimating the overall proportion of correctly classified task-selection and task-execution failures across the entire experimental session.

## The role of inhibition and associative learning in task switching

While the first aim of this study was to compare the different methodologies of disentangling task-selection failures and task-execution failures in task switching, the second aim was to investigate whether these different error types differentially affect inhibitory control in task switching, building up on a previous study from our lab (Moretti et al., 2021).

In task-switching studies, the most common measure of the processes underlying cognitive flexibility are the task-switch costs: When comparing performance between repeat trials, where the task is the same as in trial *N*-1, and switch trials, where the task is different compared to trial N-1, task-switch costs are typically observed (Roger & Monsell, 1995; Meiran, 1996). There is nowadays some consensus on the fact that performance costs associated with switching are, at least partly, due to interference caused by carryover activation of the previously relevant task-set (Allport & Wylie, 2000; Allport et al., 1994; Rubinstein et al., 2001). The automatic tendency to repeat the positively primed previous task is counteracted in switch trials by cognitive control mechanisms that inhibit the now irrelevant task-set, a presumed mechanism known as backward inhibition (Mayr & Keele, 2000; for a review, see: Koch et al., 2010). One well-established finding generally interpreted as supporting the existence of backward inhibition are N-2 repetition costs (Gade et al., 2014; Kiesel et al., 2010; Mayr & Keele, 2000). When asking participants to switch between three tasks, and the task switches on every trial, only two kinds of triplet task sequences are possible. Either after a first switch one needs to switch back to the first executed task (e.g. ABA), or three different tasks are performed in a row (e.g. CBA). The only difference between the two triplets is that in the first case the task in trial N-2 matches the one in trial N, whereas in the latter scenario it does not. The ABA kind of sequences is, therefore, referred to as N-2 repetition sequences, whereas a CBA sequence would be an N-2 switch sequence. N-2 repetition costs are the performance decrement found in the N trial of an N-2 repetition sequence, compared to the N trial of an N-2 switch sequence (Mayr & Keele, 2000; Koch et al., 2010; for a computational account see Sexton & Cooper, 2017). The most common explanation for this finding is that in an ABA sequence, task A needs to be inhibited in trial N-1 to successfully execute task B, and therefore strong persisting inhibition of this task impairs performance in trial N when task A is required again. In comparison, task A is not recently inhibited in a CBA sequence, and therefore its residual inhibition is smaller in trial N compared to ABA sequences. N-2 repetition costs are thus considered to be an empirical marker of inhibition in task switching.

But what exactly needs to be inhibited? What is carried over from the previous trial? One early idea in the task-switching literature is that abstract response categories relevant to one task (e.g. “odd”/ “even”) may become associated with the response-set (e.g. “left”/” right”) (Meiran, 2000; Schuch & Koch, 2004). If a task repeats, the same category-response associations can be easily used. If the task switches, however, interference is generated at the response selection stage, and inhibition is needed (Koch et al., 2010; Philipp et al., 2007; Schuch & Koch, 2003; Sinai et al., 2007). In favour of this idea, both switch costs and N-2 repetition costs were found to be absent following no-go trials in which response selection does not take place (Philipp et al., 2007; Schuch & Koch, 2003).

Based on these and other findings, Steinhauser and Hübner (2006) proposed the response-based strengthening account of task-switching (see also Philipp et al., 2007). According to this account, strengthening of task sets (including strengthening of category-response rules) takes place during response execution in an automatic fashion, outside the reach of cognitive control. The crucial prediction of this response-based strengthening account is that if the irrelevant task is executed in a given trial, this task is automatically strengthened so that it is the wrongly executed task that is still active in the subsequent trial. As such, trials following *task-confusion errors* are not expected to exhibit switch costs because “switching” actually requires to perform the currently activated task in these trials (and “repeating” involves to actually inhibit the currently activated task). If task selection is accomplished correctly instead, and the relevant task is executed, it is predicted that task-switch costs will be found in the following trial, irrespective of whether the correct response was chosen. In other words, task-switch costs should still be present following correct trials, and following *response-confusion errors*.

These predictions were tested in a series of experiments using bivalent task-response mappings and both congruent and incongruent trials, as described above (Steinhauser & Hübner, 2006, 2008). Switch benefits were indeed found following an error. Importantly, this was the case only if the error was made on an incongruent trial, where task-selection failures were found to be more prevalent than on congruent trials (as estimated by MPT modelling). Furthermore, similar findings were observed in subsequent studies using both univalent task-response mappings (Desmet et al., 2012) and using three response options per task (Steinhauser et al., 2017): task-switch costs were observed after response-confusion errors, but not after task-confusion errors.

### Evidence for a slow error-correction mechanism in task switching

As explained in the previous section, the response-based strengthening account has received empirical support from a number of studies that assessed task-switch costs when switching between two different tasks. Using the same logic, the response-based strengthening account also predicts that N-2 repetition costs should be reduced following a task-confusion error in trial N-2. Let us take an ABA sequence as an example. If task A in trial N-2 is not executed, because task C is instead erroneously selected, task C, and not task A, will be inhibited in trial N-1. As such, no inhibition will need to be overcome in trial N, and no N-2 repetition costs will be observed.

In a recent study using only incongruent stimuli, we found that N-2 repetition costs were indeed absent after N-2 (task-confusion) errors, but only when the response in the N-1 trial belonged to the fast part of the RT distribution (Moretti et al., 2021). In other words, if a task-confusion error was followed by a relatively fast response, N-2 repetition costs were abolished in the N trial. Otherwise, they were still observed. A very similar observation was made by Steinhauser and Hübner (2008), who found that the switch facilitation effect following a task-confusion error was present only if the post-error trial was fast. These results were interpreted as indicating the existence of a cognitive control mechanism aimed at correcting the automatic and maladaptive strengthening of the erroneously executed task set. Importantly, such a control mechanism would be slowly building (see also: Ridderinkhof, 2002; Ridderinkhof et al., 2004; Wildenberg et al., 2010), so that its corrective effects can be observed only in slow post-error trials, where it restores switch costs. Similarly to what was observed with switch costs, inhibiting the erroneously executed task-set in trial N-2 and/or re-instantiating the relevant task in trial N-2 would also restore N-2 repetition costs, thus explaining our finding of reduced N-2 repetition costs following an error only for the N-1 Fast condition.

## The present study

In the present study, we aim at further investigating this empirical signature of a slowly building error correction mechanism after task-confusion errors. Differently from our previous study, in which only incongruent stimuli were present, under the assumption that most errors would be due to task-selection failures, here we used the three different methodologies for disentangling errors due to task-selection and task-execution failures introduced earlier on. Figure 1 provides an overview of the methodologies used in each experiment. In Experiment 1, a univalent task-response mapping was employed, which provides the most intuitive way to disentangle task selection and task execution errors (Desmet et al., 2012; Meiran & Daichman, 2005). In Experiment 2, a bivalent set-up with congruent and incongruent stimuli was used, as it was used in the first studies testing the response-based strengthening account (Steinhauser & Hübner, 2006). Finally, in Experiment 3 we used the more recent methodology of presenting stimuli with a lower number of stimulus dimensions (two) than response alternatives (three) (Steinhauser & Gade, 2015; Steinhauser et al., 2017; Steinhauser & Steinhauser, 2019). Experiments 1 and 2 were pre-registered laboratory experiments; for these experiments, we pre-specified outlier criteria, design, and ANOVA approach for data analysis; we did not pre-register the addition of Bayes Factors to the ANOVA analysis, and neither did the use of MPT modelling. Experiment 3 was an online experiment. While Experiments 1 and 2 employed a similar paradigm as that of a previously published study (Moretti et al., 2021) and were carried out in the lab, Experiment 3 had a different set-up, which we expected to lead to a larger variability in the data. As this might require different outlier exclusion criteria, this experiment was not pre-registered.

Data analysis for each experiment proceeded in two steps. In a first step, we established whether the error trials classified as task-confusion errors actually contained more task-selection failures than the error trials classified as response-confusion errors. To this end, we used MPT models (Batchelder & Riefer, 1999; Riefer & Batchelder, 1988) in Experiments 2 and 3 (see right panels of Fig. 1). For Experiment 1, this was not possible by design, so we only report descriptive statistics for each error type.

In a second step, we analysed how N-2 repetition costs are modulated by the different error types, and by response speed in the N-1 trial. As outlined above, we expected N-2 repetition costs to be reduced after N-2 task-confusion errors, but not after N-2 response-confusion errors. Moreover, we expected the reduction of N-2 repetition costs after N-2 task-confusion error to be restricted to the fast N-1 trials, where a slow error-correction mechanism has not yet become effective. In contrast, no modulation of N-2 repetition costs by N-1 speed was expected for trials following N-2 response-confusion errors. To anticipate the results, with the exception of Experiment 2, we did not obtain enough trials for some of the error-type conditions: in Experiment 1, we did not obtain enough N-2 task-confusion errors; in Experiments 3, we did not obtain enough N-2 response-confusion errors. In these experiments, we, therefore, analysed N-2 repetition costs in a simplified design with just two levels of the N-2 error type factor (Experiment 1: N-2 correct vs. N-2 response-confusion error; Experiment 3: N-2 correct vs. N-2 task-confusion error).

## General method

### MPT modelling

MPT are a class of statistical models fitted on frequency data that allow estimating the probability of an observed response to be produced by the occurrence of a series of underlying cognitive events (Batchelder & Riefer, 1999; Hu & Batchelder, 1994; Riefer & Batchelder, 1988). In our case, we used MPT models to estimate the probability of a given response to be produced by the success or failure of task-selection and response-selection processes. The right panels of Fig. 1 represent examples of such models. As can be seen, in MPT models the probability of a cognitive event to succeed is represented by a parameter θ_s_ whose value is constrained to fall between 0 and 1. An observable event (e.g. an error that is empirically categorized as task-confusion error) is considered to be the result of the success (or failure) of those unobservable cognitive events. Crucially, the structure of a model depends on the cognitive processes that the experimenter assumes to take place in the paradigm. For this reason, the equations describing an MPT model depend on how a response is thought to arise from the specific cognitive mechanisms involved in the paradigm. Having set the equations for mapping the observable response categories to the cognitive processes, and knowing the frequencies with which each response category is observed, it is possible to estimate the probabilities of the latent cognitive events, which are represented by the model’s parameters (Hu & Batchelder, 1994). After this, the probability of occurrence of an observable event can be computed by first multiplying the model’s parameters along a branch, and second, adding the resulting probabilities for all branches that lead to this event.

In our case, MPT can be fit to estimate the probability that an observed error category would be produced depending on the outcome of task selection and response selection processes. Figure 1 illustrates how latent cognitive events are mapped to overt response categories in our experiments. The set of Eqs. 1 and 2 provide a mathematical formalisation of such models. In our models, we defined a parameter T, representing the probability of correct task selection, and a parameter R, representing the probability of a correct response selection for the chosen task. For the incongruent trials in Experiments 2 and 3, we also defined a third parameter RE (random execution), representing the probability of randomly choosing the correct response after a combined task-selection failure and response-selection failure had occurred (see Fig. 1). As we assumed this to be a random process of selecting one of two possible responses, RE was set to 0.5. For Experiment 2, the observed response categories were “correct incongruent” “error incongruent” (corresponding to task-confusion errors), “correct congruent”, and “error congruent” (corresponding to response-confusion errors). For experiment 3, the observed response categories were “correct”, “task-confusion error”, “response-confusion error”. Models were fit to each individual participant in R using the MPTinR package (Singmann & Kellen, 2013).

Once the model’s parameters had been estimated, we assessed the capacity of each methodology to correctly classify the unobservable cognitive event of a task-selection failure into the empirical category of “task-confusion errors”, by computing two indices. First, we computed the estimated percentage of task-selection failures among classified task-confusion errors. We did so by dividing the probability that an observed task-confusion error resulted from a task-selection failure (i.e. considering only those MPT branches that include parameter (1 − *T*) and lead to a task-confusion error) by the total probability of observing a task-confusion error (i.e., considering all MPT branches that lead to a task-confusion error). Second, we computed the estimated percentage of task-selection failures among classified response-confusion errors, by dividing the estimated probability that a response-confusion error would be observed given a task-selection failure (i.e. considering only those MPT branches that include parameter (1 − *T*) and lead to a response-confusion error) by the total probability of observing a response-confusion error (i.e., considering all MPT branches that lead to a response-confusion error).

### Analysis of N-2 repetition costs

In all experiments, N-2 repetition costs were analysed in an ANOVA design, with Task Sequence (N-2 repetition, N-2 switch), N-2 Accuracy (N-2 correct, N-2 error) and N-1 speed (fast vs. slow, as defined by median split) as independent variables.

Additional ANOVAs with only Task Sequence and N-2 Accuracy as independent variables are reported in the Appendix. While in the main text we only analyse N-2 repetition costs in RTs, the additional ANOVAs reported in the Online Appendix were performed on both RTs and arcsine transformed error rates as dependent variables.

The different error types were defined somewhat differently in the three different paradigms: in Experiments 1 and 3, the N-2 Accuracy factor included three levels (N-2 correct, N-2 task-confusion error, N-2 response-confusion error). In Experiment 2, the design included N-2 Congruency as an additional independent variable (N-2 congruent, N-2 incongruent); in this experiment, errors in congruent trials would be classified as response-confusion errors, whereas errors in incongruent trials would be labelled as task-confusion errors.

If we did not obtain enough trials for reliable estimation of performance in one of the N-2 error conditions, we would remove this condition from statistical analysis and proceed with a simplified design. Significant 3-way interactions in the ANOVA involving the N-1 Speed factor were further explored by dividing the dataset into two separate N-1 fast and N-1 slow datasets, and performing a 2-way ANOVA on each of those. 2-way interactions were further analysed with post-hoc paired *t*-tests, assessing N-2 repetition costs separately in the different N-2 error conditions.

The dependent variable of the ANOVAs was mean RT (see Online Appendix for ANOVAs on arcsine transformed error rates). In all ANOVAs, we report both ${\eta }_{p}^{2}$ and ${\eta }_{G}^{2}$ to provide useful measures of effect size for both power analysis and meta-analysis, respectively (Bakeman, 2005; Lakens, 2013; Olejnik & Algina, 2003). Cohen’s *d*_*z*_ is reported for paired t-tests, computed by dividing mean difference scores by their standard deviation (Brysbaert, 2019; Lakens, 2013).

### Computation of Bayes factors (BFs)

In addition to frequentist ANOVAs, we also report Bayes factors (BFs) for most of the effects under consideration. In particular, we computed BFs for all the ANOVAs involving less than 4 independent variables. This choice was made for two reasons. First, it is computationally very demanding to compute BFs for such complex designs (see below for the computation procedure). Second, we used BFs mostly for assessing evidence in favour of the null hypothesis: as we do not make any prediction for a null effect in any 4-way ANOVA, computation of BFs was not strictly needed for our purposes.

BFs were calculated in two steps. First, for each set of independent variables models were built with all the possible combinations of effects involving them^1^ (e.g., for a design with the independent variables A and B, the following models were built: Null model without any effects, model with only main effect of A, model with only main effect of B, model with main effects of A and B, but no interaction, full model with main effects of A and B and interaction AxB). After the models were built, and the likelihood for each model given the data were computed, BFs were calculated as the ratio between this likelihood and that of a null model containing only the grand average and participants as a random effect. Afterwards, inference on a particular effect of interest was achieved by comparing the BF of the best fitting model (i.e. that with the highest BF compared to the null) with that of an identical model which differs only in the presence/absence of the effect of interest. For example, if the best fitting model contains the main effect of A and an AxB interaction, evidence in favour of the interaction was computed as the ratio between this model’s BF and the BF of a model only containing the main effect of A (Rouder et al., 2017).

### Data trimming

For both MPT analysis and analysis of N-2 repetition costs, in all experiments, the first two trials of each block were removed, along with the trial immediately following an error, timeouts, and the subsequent two trials. Fast guesses were defined as responses below 300 ms in the laboratory Experiments 1 and 2, whereas in the online Experiments 3, we used a threshold of 100 ms as responses were much faster in those experiments. Fast guesses and the subsequent two trials were removed in each experiment. For RT analysis of N-2 repetition costs, error trials were also removed. After applying these trimming criteria, data from all participants holding less than 10 trials in any of the ANOVA conditions (Task Sequence x N-2 Accuracy x N-1 Speed) were excluded. Furthermore, data from participants that made systematic use of fast guesses (i.e. > 10% of the total number of trials), or whose error rate was above 33% were excluded from the analysis.

## Experiment 1

The first method used in the literature for disentangling errors due to task selection and task execution failure was to use univalent task-response mappings (Desmet et al., 2012; Meiran & Daichman, 2005; Meiran et al., 2001), where separate response sets are used for the different tasks.

### Methods

#### Pre-registration

Experiment 1 was pre-registered at https://aspredicted.org #27,335 (https://aspredicted.org/v7qm9.pdf).

#### Participants

30 participants (25 females) spanning 18–25 years of age (20.9 ± 2.2) took part in the study at the Institute of Psychology at RWTH Aachen, in exchange for course credits.

#### Stimuli

The employed stimuli were colored geometrical shapes presented at different orientations on a grey background. Each stimulus dimension varied on two levels, each mapped to a different key. The shape dimension could be a rectangle or triangle. Colors could be red or blue. The orientation of the figure could be upright, or horizontal. The total area subtended by the visual stimuli was roughly equal. From a viewing distance of 100 cm, the base and the height of the rectangle were 11.42° and 5.72°, respectively. The isosceles triangle had a base of 6.86° and a height of 13.12°.

A cue presented at the beginning of each trial indicated which stimulus dimension was to be attended (for further details, see the *procedure* section). The letters “A”, “B” and “C” were used as cues. Each cue was 0.85° high and 0.5° wide. The experiment was run using PsychoPy toolbox 3.1 (Peirce et al., 2019).

#### Trial procedure

On each trial participants classified, via a button press, a visual stimulus according to one of three possible rules or tasks. The currently relevant task was indicated at the beginning of the trial by a cue. If letter “A” was presented, participants had to indicate the stimulus’ shape (*shape classification task*). If the cue was “B”, the stimulus’ color had to be recognized (*color classification task*). Finally, the “C” cue indicated to classify the stimulus according to its orientation (*orientation classification task*).

For responding, participants used 6 keys: ‘X’, ‘C’,’V’,’B’, ‘N’, ‘M’ of a QWERTZ keyboard. Each task was mapped to two different keys to be pressed with one finger of each hand. For instance, the color task could be mapped to keys ‘V’ and ‘B’ to be pressed with the left and right index finger, respectively. This way we aimed at distinguishing between task-confusion errors and response-confusion errors. A response was labelled as a task-confusion error whenever the correct response for one of the wrong task sets was executed (i.e., 2 of the 6 keys corresponded to task-confusion errors for each stimulus). Response-confusion errors were present when the participant pressed a key relevant to the present task, but inappropriate for the stimulus at hand (1 of the 6 keys corresponded to a response-confusion error for each stimulus). The remaining errors were considered mixed errors (i.e., the remaining 2 of the 6 keys).

Each trial started with a cue being presented for 100 ms. After this time elapsed, the cue disappeared and the screen stayed blank for 300 ms, followed by a stimulus presentation. The target then stayed on the screen until participants’ response, up to a maximum of 2000 ms. If no response was given within this interval, the trial was considered a timeout. Either after this time, or after a response, the screen turned blank again, and a new trial started after 100 ms.

#### Experimental procedure

The experiment began with a practice phase consisting of 3 pure blocks and 4 mixed blocks. In pure blocks, participants performed only one task in isolation for 48 trials. As the main aim of pure blocks was to get familiarised with the S-R mappings, there was a pure block for each task. Furthermore, throughout practice, and contrary to the experimental phase, a sheet with the S-R mapping was attached 20 cm above the computer monitor, to further aid memorization. Finally, only during practice, feedback was presented following error and timeout trials. After the pure blocks, participants were introduced to the practice mixed blocks, in which all tasks were present in alternation. Before the practice of mixed blocks started, it was communicated that the three fastest participants whose error rate would not exceed 25%, would receive a monetary award (20€, 10€ and 5€ for the first, second and third prize, respectively). In this context, it was emphasised that whenever the error rate would fall under 15%, the participant should speed up. On the other hand, if errors were above 20%, it was recommended to slow down. To enable participants to monitor their own performance, the mean error rate and mean response time were displayed at the end of each block. Even though it was made explicit that performance in the practice mixed blocks would not be taken into account for determining the winners of the monetary award, speed and accuracy feedback were provided at the end of each block in this phase as well. Four practice mixed blocks of 120 trials each were presented to ensure stable performance.

After these, the experimental phase began, consisting of 18 mixed blocks of the same length, for a total of 2160 trials. Across participants, stimuli and tasks were presented in a pseudorandom fixed sequence, which met the following constraints. Only task switches were present in the experiment (i.e., the task could never repeat). Furthermore, the number of N-2 repetition sequence and N-2 switch sequence was equal within each block, as well as for each combination of task and stimuli. Finally, to avoid N-2 repetition costs to be confounded with episodic repetition and retrieval effect (Gade et al., 2017; Grange et al., 2017; Kowalczyk & Grange, 2019), a stimulus presented in trial N could never appear in *N* + 1 or *N* + 2 trials.

#### Design

For the N-2 repetition costs analysis we planned to run a 2 × 3 × 2 ANOVA with factors Task Sequence (N-2 repetition, N-2 switch), N-2 Accuracy (correct, task-confusion error, response-confusion error), and N-1 Speed (fast, slow) as independent within-subjects’ variables, and RTs as a dependent variable. As, following data trimming, there were not enough trials in the N-2 task-confusion error condition, this level was removed and we proceeded with a simplified 2 × 2 × 2 design. With such a design we did not expect to find any modulation of N-2 Accuracy or N-1 Speed on the N-2 repetition costs. To confirm the absence of a 3-way interaction (which we only would have expected in the full design with the condition of N-2 task-confusion errors included), we computed BFs for the simplified 3-way ANOVA.

### Results

#### Data trimming

Data trimming proceeded according to the criteria specified in the General Method. No participant had to be excluded on the basis of the pre-defined criteria for excessive fast guesses or due to low accuracy. When re-assessing the number of trials per participant in each cell of the 2 × 2 × 2 ANOVA design, six subjects still did not meet the criterion of at least 10 trials in the N-2 response confusion conditions. Consequently, their data were removed from further analysis, leaving a sample of 24 participants.^2^

#### Frequency of different error types

After data trimming, participants made, on average, an error in 7.4% of the total number of trials (SD 3.8%, range 3.0–14.8%). 79.8% of these error trials were classified as response-confusion errors (SD 8.6%, range 56.0–93.5%), 14.8% as task-confusion errors (SD 7.6%, range 4.8–38.0%), and the remaining 5.4% as mixed errors (SD 2.9%, range 0.5–12.4%).

#### Proportion of task-selection failures and task-execution failures

The model for Experiment 1 is depicted in Fig. 1. Note that for Experiment 1, we could not fit an MPT model, because each possible response category is uniquely linked to one processing path. Therefore, we simply computed the probability of task-selection failures per participant as the sum of task-confusion errors and mixed errors, divided by the total number of trials. The probability of a task-selection failure in Experiment 1 (which corresponds to parameter (1 − *T*) in the MPT models of Experiments 2 and 3) was 1.5% (SD 0.9%, range 0.3–3.7%). Likewise, the probability of a task-execution failure in Experiment 1 was computed as the sum of response-confusion errors and mixed errors, divided by the total number of trials. The probability of a task-execution failure in Experiment 1 (which corresponds to parameter (1 − *R*) in the MPT models of Experiments 2 and 3) was 6.3% (SD 3.4%, range 2.4–12.9%).

#### Analysis of N-2 repetition costs

**Fig. 2:**
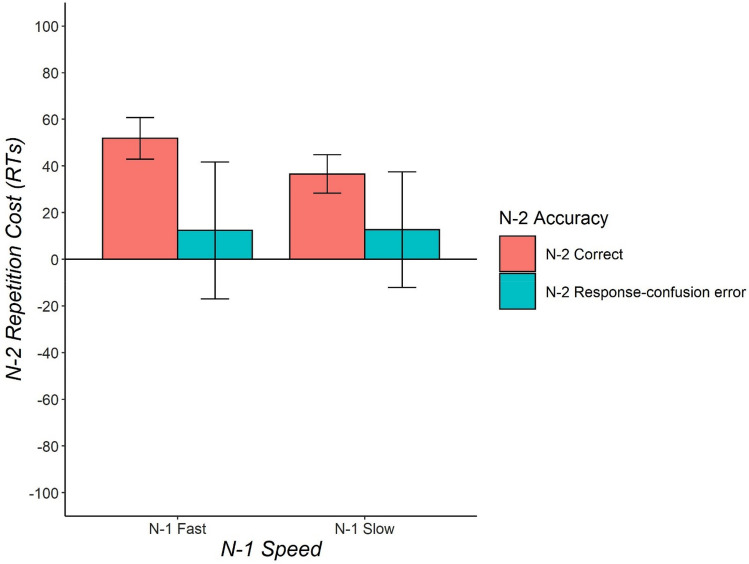
Experiment 1: Mean N-2 repetition costs (in ms) as a function of N-2 Accuracy (N-2 correct, N-2 response-confusion error), and N-1 Speed (N-1 slow, N-1 fast, as defined by median split on N-1 reaction times). Error bars indicate one standard error of the mean

Descriptive statistics are reported in Fig. 2. N-2 repetition costs were found overall (28 ms) as demonstrated by a main effect of Task Sequence, *F*(1,23) = 6.72, *p* = 0.016, ${\eta }_{p}^{2}$ = 0.23, ${\eta }_{G}^{2}=0.012$, *BF*_*10*_ = 5.75. Furthermore, there was a main effect of N-1 Speed, *F*(1,23) = 5.33, *p* = 0.030, ${\eta }_{p}^{2}$ = 0.19, ${\eta }_{G}^{2}=0.010$, *BF*_*10*_ = 2.90, indicating that trials following a slow response were slower compared to trials following a fast response. N-2 Accuracy significantly interacted with N-1 Speed, *F*(1,23) = 11.02, *p* = 0.003, ${\eta }_{p}^{2}$ = 0.32,${\eta }_{G}^{2}=.012$, *BF*_*10*_ = 6.33. Most importantly, the three-way interaction between Task Sequence, N-2 Accuracy and N-1 Speed was not significant, *F* < 1, *BF*_*10*_ = 0.31. N-2 repetition costs following an N-2 correct response were 52 ms for the N-1 fast condition and 37 ms for the N-1 slow condition. If there was a response-confusion error in N-2, N-2 repetition costs were identical irrespective of N-1 Speed (13 ms in both N-1 fast condition and N-1 slow condition).

### Discussion

Using separate response sets for each task, we aimed at disentangling task-selection failures (here: choosing a response from the wrong response set) from task execution failures (here: choosing a response from the correct response set, which however does not map to the relevant dimension of the stimulus). The results showed that participants produced a sufficient number of empirically classified response-confusion errors (about 6.3% on average), but hardly made any empirically classified task-confusion errors (about 1.5% on average). It is unlikely, however, that participants produced so few task-selection failures in this paradigm. Possibly, failures in task selection were wrongly classified as “response-confusion errors”. In particular, the task cue might have led to a strong pre-activation of the task-specific response-set, so that participants are more likely to produce a response from this pre-activated response-set than from the alternative response-set. However, this does not necessarily imply that the participants are actually attending to the task-relevant stimulus dimension, and hence, are performing the correct task (see Meiran & Daichman, 2005, for a discussion of task preparation vs. effector preparation in switching between tasks with separate response sets).

Regarding N-2 repetition costs, we expected to find reduced N-2 repetition costs after N-2 task-confusion errors, but not after N-2 response-confusion errors. Moreover, we expected the reduction of N-2 repetition costs after N-2 task-confusion errors to be restricted to those trials with a little post-error slowing in the N-1 trial (i.e., the fast N-1 trials), where the slow error-correction mechanism has not yet kicked in (see Moretti et al., 2021). However, we did not obtain enough task-confusion errors for statistical analysis, and therefore we could only compare the conditions with N-2 response-confusion errors and N-2 correct trials. Under such circumstances, we predicted that the three-way interaction of N-2 accuracy, Task Sequence, and N-1 Speed would not be present. In line with our expectation, we indeed did not observe any three-way interaction (*F* < 1, BF_10_ = 0.31). N-2 repetition costs after N-2 response-confusion errors were numerically identical after fast and slow N-1 trials, suggesting that N-2 repetition costs after N-2 response-confusion errors were not modulated by N-1 speed (for further analysis excluding the N-1 speed factor, see the Online Appendix).

## Experiment 2

The paradigm employed in Experiment 1 (using a univalent task-response mapping) failed to elicit enough task-confusion error trials, possibly due to the strong pre-activation of effectors during the task-preparation interval (CSI). In Experiment 2, we used a methodology similar to that employed by Steinhauser and Hübner (2006). We employed both congruent and incongruent stimuli in a bivalent task-response setting, and assumed that errors in incongruent trials were often due to task-selection failure, while errors in congruent trials would be attributable to task-execution failure. To check whether this assumption held to be valid, we used MPT modelling. We predicted that N-2 repetition costs would be significantly reduced following an N-2 error compared to N-2 correct sequences, but only if the N-2 trial was incongruent (and hence, task-selection failures occurred), and only when N-1 trial RT was fast (and hence, no task-level correction mechanism has kicked in). In contrast, N-2 repetition costs following an error in an N-2 congruent trial (where task-execution failure occurred) was not expected to be influenced by N-1 trial speed.

### Methods

#### Pre-registration

Experiment 2 was pre-registered at: https://aspredicted.org/6ut4t.pdf.

#### Participants

60 participants (34 females) spanning 18–34 years of age (24 ± 4.1) took part in the experiment at the Institute of Psychology at RWTH Aachen, in exchange for money (12 €) or course credits.

#### Stimuli

**Fig. 3:**
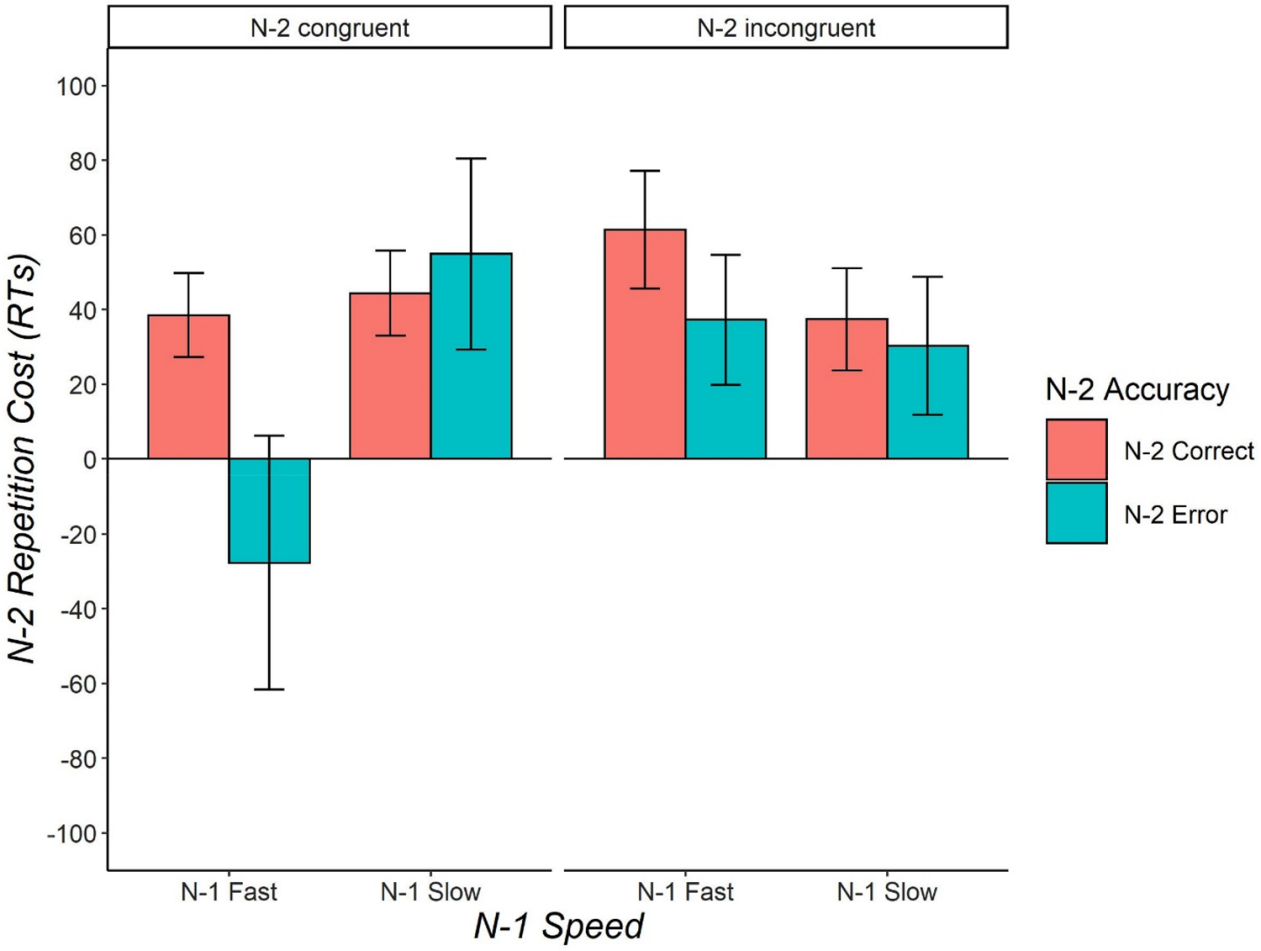
Experiment 2: Mean N-2 repetition costs (in ms) as a function of N-2 Accuracy (N-2 correct, N-2 error), N-2 Congruency (N-2 congruent, N-2 incongruent), and N-1 Speed (N-1 slow, N-1 fast, as defined by median split on N-1 reaction times). Error bars indicate one standard error of the mean. According to the experimental logic, errors in congruent trials correspond to response-confusion errors, while errors in incongruent trials correspond to task-confusion errors

Target stimuli were identical to those used in Experiment 1, except for two differences. First, both congruent and incongruent stimuli were used. Second, the three stimulus dimensions varied on three levels instead of two. In addition to rectangle and triangle, an oval was also present covering roughly the same visual area as the other shapes. The color green was added to red and blue. Finally, the figures could be presented also tilted by 45° in addition to vertically or horizontally. The choice of adding a third level for each stimulus dimension was made to obtain incongruent trials in which the correct response to each dimension was mapped to a different key, which, with three tasks, requires the presence of three keys. Similarly, stimuli were defined to be congruent only when the correct response to each task afforded by the stimulus was mapped to the same key. The stimulus-set was thus composed of 6 figures, 3 congruent and 3 incongruent (Fig. 3).

#### Responses

For responding, the C, V and B keys of a QWERTZ keyboard were used. One key was always mapped to rectangle, red or vertical, another one was used for triangle, blue or horizontal, and the third key indicated oval, green or tilted. The mapping of each of these three combinations (e.g. rectangle-red-vertical) to a particular key (C, V, or B key) was fully counterbalanced across participants, resulting in 6 different stimulus–response (S-R) mappings.

#### Procedure

The procedure was similar to that of Experiment 1. However, due to the different number of stimuli employed, each block was composed of 108 trials instead of 120. To render the total trial number identical across experiments, the number of blocks for the experimental phase was increased from 18 to 20 (total number of trials: 2160).

#### Design

We performed a 2 × 2 × 2 × 2 within-subjects ANOVAs on RTs, with Task Sequence (N-2 repetition, N-2 switch), N-2 Accuracy (N-2 Correct, N-2 Error), N-2 Congruency (N-2 Congruent, N-2 Incongruent), and N-1 Speed (Fast, Slow), as factors. For the same design excluding N-1 Speed, on both RTs and error rates, the reader is referred to the Appendix. We expected a 4-way interaction indicating that for N-2 Incongruent, but not for N-2 Congruent trials, N-2 repetition costs would be modulated by N-2 Accuracy and N-1 Speed. That is, we expected to observe a 3-way interaction for N-2 Incongruent trials, but no such 3-way interaction for N-2 Congruent trials. We, therefore, conducted follow-up 3-way ANOVAs, including BFs, separately for the N-2 Incongruent and N-2 Congruent conditions.

#### Data trimming

Participants who made systematic use of fast guesses (i.e. > 10% of the total number of trials) (*N* = 4), and/or who had an accuracy below 33% (*N* = 4) were excluded from the analysis. Moreover, participants who had less than 10 trials per condition of the ANOVA design (Task Sequence x N-2 accuracy x N-2 congruency x N-1 Speed) after data trimming were excluded. This led to exclusion of a large number of participants (*N* = 31), mainly driven by the fact that these participants did not have enough trials in the N-2 congruent error conditions. The analyses were therefore conducted on a total of 21 participants.

### Results

#### Frequency of different error types

On average, participants made an error in 17.2% of the trials (SD 4.9%, range 8.6–29.0%). Errors in congruent trials were on average 5.7% of the total (SD 2.6%, range 2.4–12.6%), whereas errors in incongruent trials were 11.5% of the total (SD 3.3%, range 6.2–19.2%).

#### Proportion of task-selection failures and task-execution failures

In Experiment 2, we assumed that errors in incongruent trials are more likely to be due to failure in task selection than errors in congruent trials. To test this assumption empirically, we modelled the error data using MPT. A visual representation of our model can be found in Fig. 1. The set of equations describing such a model was as follows:

1 $$ \begin{gathered} p\left( {\text{correct congruent}} \right) \, = \, T*R \, + \, \left( {1 - T} \right)*R \hfill \\ p\left( {\text{error congruent}} \right) \, = \, T*\left( {1 - R} \right) \, + \, \left( {1 - T} \right)*\left( {1 - R} \right) \hfill \\ p\left( {\text{correct incongruent}} \right) \, = \, T*R \, + \, \left( {1 - T} \right)*\left( {1 - R} \right)*0.5 \hfill \\ p\left( {\text{error incongruent}} \right) \, = \, T*\left( {1 - R} \right) \, + \, \left( {1 - T} \right)*R \, + \, \left( {1 - T} \right)*\left( {1 - R} \right)*0.5 \hfill \\ \end{gathered} $$

The mean estimated parameters were *R* = 0.885 (SD = 0.05, range 0.778–0.949), and *T* = 0.864 (SD = 0.07, range 0.748–1). After estimating the models’ parameters, we estimated the number of task-selection failures among classified task-confusion errors (i.e. among errors in incongruent trials), and the number of task-selection failures among response-confusion errors (i.e. among errors in congruent trials). This was done separately for each participant using the following equations:

2 $$ \begin{gathered} p{(} {\text{task-selection failure}} {|} {\text{error incongruent}}) = \frac{{\left( {1 - T} \right)*R + \left( {1 - T} \right)*\left( {1 - R} \right)*0.5}}{{T*\left( {1 - R} \right) + \left( {1 - T} \right)*R + \left( {1 - T} \right)*\left( {1 - R} \right)*0.5}} \hfill \\ p{\text{(task-selection failure}} {|} {\text{error congruent}}) = \frac{{\left( {1 - T} \right)*\left( {1 - R} \right)}}{{T*\left( {1 - R} \right) + \left( {1 - T} \right)*\left( {1 - R} \right)}} \hfill \\ \end{gathered} $$

The probability that an error in an incongruent trial was actually due to task-selection failure was on average 54.8% (SD = 20.3). At the same time, the probability that an error on a congruent trial was due to task-selection failure was estimated to be 13.5% (SD = 6.8%). According to these estimates, therefore, the proportion of task-selection failures in incongruent errors was roughly 4 times higher than in congruent errors. Our assumption that errors in incongruent trials are more likely to be due to task-selection failures than errors in congruent trials was thus corroborated.

#### Analysis of *N*-2 repetition costs

Descriptive statistics for the 4-way ANOVA design are reported in Fig. 2. A significant main effect of Sequence, *F*(1,20) = 22.99, *p* < 0.001, ${\eta }_{p}^{2}$ = 0.53,${\eta }_{G}^{2}=0.015$, indicated the overall presence of N-2 repetition costs (34 ms). Furthermore, the main effect of N-1 Speed was also significant, *F*(1,20) = 17.91, *p* < 0.001, ${\eta }_{p}^{2}$ = 0.47,${\eta }_{G}^{2}=0.040$, indicating that after a slow response in N-1, responses were slower than after a fast response (54 ms). Also, we observed a significant 3-way interaction of the factors Task Sequence, N-2 Congruency, and N-1 Speed, *F*(1,20) = 4.78, *p* = 0.041, ${\eta }_{p}^{2}$ = 0.19,${\eta }_{G}^{2}=0.003$, as well as a marginally significant 3-way interaction between Task Sequence, N-2 Accuracy, and N-1 Speed, *F*(1,20) = 3.84, *p* = 0.064, ${\eta }_{p}^{2}$ = 0.16,${\eta }_{G}^{2}=0.002$. The four-way interaction was not significant, *F*(1,20) = 1.42, *p* = 0.247, ${\eta }_{p}^{2}$ = 0.06,${\eta }_{G}^{2}<0.001$. No other effect was significant.

For exploratory purposes, we analysed congruent and incongruent trials separately. In the N-2 congruent dataset, a significant 3-way interaction between Task Sequence, N-1 Speed and N-2 Accuracy was found in the frequentist ANOVA, *F*(1,20) = 4.59, *p* = 0.045, ${\eta }_{p}^{2}$ = 0.19,${\eta }_{G}^{2}=0.004$, but was not supported by the Bayes factor, BF_10_ = 0.73. Numerically, the data pattern was as follows: in N-1 fast trials, N-2 repetition costs were present following a correct response in N-2 (38 ms), *t*(20) = 3.42, *p* = 0.003, *d*_*z*_ = 0.75, BF_10_ = 15.26, but turned into a numerical facilitation in the N-2 error condition (− 28 ms), *t* > − 1, BF_10_ = 0.31. In contrast, in N-1 slow trials, N-2 repetition costs were present both after N-2 correct (44 ms), *t*(20) = 3.90, *p* < 0.001, *d*_*z*_ = 0.85, BF_10_ = 40.10 and after N-2 error trials (55 ms), *t*(20) = 2.14, *p* = 0.044, *d*_*z*_ = 0.47, BF_10_ = 1.49. In the N-2 incongruent dataset, on the other hand, the 3-way interaction was not significant, *F* < 1, *BF*_*10*_ = 0.29. N-2 repetition costs after N-1 fast trials were 61 ms after N-2 correct and 37 ms after N-2 error; N-2 repetition costs after N-1 slow trials were 37 ms after N-2 correct and 30 ms after N-2 error.

### Discussion

In Experiment 2, we wanted to distinguish errors due to task-selection failure and task-execution failure by mapping them to errors occurring on incongruent and congruent trials, respectively. We report, in line with previous studies (Meiran & Daichman, 2005; Steinhauser & Hübner, 2006), that this methodology does allow us to distinguish between the two sources of errors, with task-selection failures being present almost four times more often among errors in incongruent trials (53.5% of the occurrences) than among errors in congruent trials (13.5% of the occurrences).

Having validated our assumptions on the mapping between observable response categories and latent cognitive processes in our paradigm, we went on to further test whether N-2 repetition costs are affected by either of the two error types in N-2. In line with the response-based strengthening account (Steinhauser & Hübner, 2006, 2008), we predicted N-2 repetition costs to be abolished following errors in incongruent trials, but to be spared when following errors in congruent trials. Most importantly, this would hold only for the fast N-1 condition. The results, however, did not reveal the predicted four-way interaction. When analyzing N-2 repetition costs separately in the different conditions, the data pattern was not as expected. On the one hand, trials following an N-2 congruent error showed a reduction in N-2 repetition costs that was modulated by N-1 speed (but only in frequentist ANOVA, not supported by BF), thus displaying the pattern that was predicted for the N-2 incongruent trials. On the other hand, N-2 incongruent trials showed no significant modulation of N-2 repetition costs by N-2 Accuracy in neither of the N-1 Speed levels.

The latter finding is particularly surprising for us when one considers that in our previous study, using the exact same paradigm, the expected pattern of results in N-2 incongruent trials was replicated in all three experiments (Moretti et al., 2021). The only differences between the previous and the present study were that, while in our previous study we used only incongruent stimuli, congruent trials were introduced here, and that, as a consequence, the experiment lasted twice as long. We consider three possibilities for explaining this discrepancy.

The first is that in our previous study, the observed effect was driven by task-execution failures that we erroneously classified as task-confusion errors. This idea may seem plausible when considering that our MPT model suggests that almost half of the errors in incongruent trials are actually due to task-execution failures. However, as will be shown in Experiment 3, our previous results could again replicate, even when the proportion of task-selection failures among classified task-confusion error was far from 100%. The second possibility is that practice effects may have influenced the recruitment of the cognitive control mechanism correcting for the automatic strengthening of the erroneously executed task-set, thus making this mechanism more efficient in the second half of the present experiment. It is indeed well established that transient adjustments in cognitive control are sensitive to global features of the task, the effect of which may take time to build up over the experiment (Bugg & Crump, 2012; Cochrane et al., 2021; Ridderinkhof, 2002Wenke et al., 2015). We will come back to this point in the general discussion. A third possibility is that introducing congruent trials per se may have led to some changes in the efficiency of the proposed cognitive control mechanism. For example, the efficiency of cognitive control in reducing conflict in task selection is increased as a function of the number of high-conflict trials in the experiment (for a recent review see: Braem et al., 2019). However, if similar effects were acting on our error-triggered cognitive control mechanism, we would expect to observe the opposite pattern, with a strong re-establishment of N-2 repetition costs following a task-confusion error in the situation where N-2 incongruent trials were most common (i.e. in our previous study).

## Experiment 3

After using a univalent task-response mapping in Experiment 1, and comparing errors in congruent and incongruent trials in Experiment 2, in Experiment 3, we come to the third methodology that has been employed in the literature, using less stimulus dimensions than response keys in a given trial (see Fig. 1).

In addition to using a third methodology, Experiment 3 was also designed so to assess the potential impact of time pressure on the occurrence of response-confusion errors. We reasoned that when little time is given both for preparing the task, and for responding to the stimulus, participants may be led to rely more on a bottom-up strategy in which “capture errors”, namely errors due to attention being captured by the distractor, would be more likely. Empirically, this would therefore result in an abundance of task-confusion errors, but only a small number of response-confusion errors. As in Experiment 2, we had to exclude a large part of the sample due to a lack of response-confusion errors, in Experiment 3 we had three experimental groups. In the first group, the same CSI and response time limit were given to the participants as in Experiment 1 and 2 (High time-pressure group). In the second group, CSI was increased from 400 to 1000 ms while the time limit was as before (Long CSI group). In the third group, the CSI was 400 ms, but the time limit for responding was removed (No response limit group). With this configuration, we aimed at assessing whether relaxing time pressure would help in eliciting more response-confusion errors in the Long CSI and No response limit groups.

### Methods

#### Participants

Experiment 3 was built and run online using Gorilla (Anwyl-Irvine et al., 2020). Participants could access the study via a link advertised online. Before clicking on the link participants were informed of the aim and duration of the study (approx. 50 min), as well as of the presence of a monetary prize based on performance. 144 participants (84 females) took part in Experiment 3 after giving informed consent (48 participants per group). Their age spanned between 19 and 30 years (23.8 ± 3.3).

#### Stimuli

Each stimulus consisted of two spatially separated elements presented to the left and right of the screen center on a white background. Each element of the pair could be a number (“1”, “5” or “9”), a letter (“A”, “M” or “Z”), or a mathematical symbol (“ < ”, “ = ” or “ > ”). Importantly, only elements of two different categories (e.g. a number and a symbol) could form a stimulus. As the task of the participants was to attend to only one of the two categories, we will refer to the element to be attended to as the target, and to the other element as the distractor. Furthermore, only incongruent stimuli were selected. Applying these constraints, the stimulus set was composed of 36 stimuli in total (i.e. 18 element pairs, in which each element would occur equally often on the left or on the right).

#### Procedure

After giving their consent, participants received the study’s link. Before running the experiment, they were required to open the link in either Safari or Chrome, as these browsers have been found to be the most precise in terms of duration of stimuli presentation and reaction times recording (Pronk et al., 2020). After demographics data were collected, the experiment began with instructions. Participants were informed that they would have to switch among three different classification tasks, indicating the identity of the presented number, letter, or math symbol. They were instructed to categorize one of the two elements making up the stimulus (i.e. the target), while ignoring the other element (i.e. the distractor). When a decision was reached, the participant should signal the target identity by pressing one of three keys. In each trial, one key was mapped to the correct response, one corresponded to the correct response for the distractor, and a third key was neither mapped to the target nor the distractor. An error was, therefore, classified as a task-confusion error if the participant pressed the correct key for the distractor, whereas if the third key was pressed, a response-confusion error was produced. Keys T, G and B on a QWERTZ keyboard were used, which are vertically arranged on the keyboard. Participants were instructed to use the ring, middle, and index finger of their left hand for responding, and place these fingers on the T, G, and B keys throughout the experiment. Importantly, the set of possible targets in each task was composed of elements that could be easily ordered e.g. “1” < “5” < “9”. This allowed creating a relatively easy stimulus–response (S-R) mapping in which the ordinal position of the element within its category was reflected in the spatial location of the key to be pressed. In this way “lower elements” (i.e. “1”, “A” and “ < ”) were mapped to the top key (i.e. T), “middle elements” (i.e. “5”, “M” and “ = ”) to the middle key (i.e. G), and highest elements (i.e. “9”, “Z” and “ > ”) to the lower key (i.e. B).

At the beginning of each trial, a cue was presented centrally on the screen for 400 ms (for the High-time pressure and the No response limit groups), or for 1000 ms (for the Long CSI group), indicating which task to perform next. Abstract symbols were used for cueing the tasks (i.e. the drawings of a sun, a moon, and a cloud), and the six possible cue-task mappings were counterbalanced across participants. Following cue presentation, the stimulus appeared centrally on the screen for 300 ms (with one element presented to the left of the screen center, the other element to the right of the screen center). After this time elapsed, participants were given 1,500 ms for providing their response. In the No response limit group, this deadline was removed. A new trial began 100 ms after a response or after the response deadline was reached.

Following instructions, participants performed three pure blocks of 15 trials each in which only one task was present throughout the block. Furthermore, another practice block of 144 trials was presented in which participants switched among the tasks: this block was identical to the test phase, except that visual feedback was presented for 1000 ms following a timeout or an incorrect response. Once practice was over, participants were informed about the monetary price as in the other experiments. The experimental phase consisted of 12 blocks of 144 trials each, for a total of 1,728 trials. In all blocks of the test phase, the task switched in every trial, and each stimulus was presented an equal number of times in the two tasks it could afford, in both N-2 switch and N-2 repetition sequences. The stimuli could not repeat from trial *N* to *N* + 1 and repeated very rarely from trial *N* to *N* + 2 (43 times in the whole experiment).

#### Design

The planned analysis design comprised a 2 × 3 × 2 × 3 mixed ANOVA with Task Sequence (N-2 repetition, N-2 switch), N-2 Accuracy (N-2 correct, N-2 task-confusion error, N-2 response-confusion error), and N-1 Speed (N-1 Fast, N-1 Slow) as within-subjects factors, and Group (High time-pressure, Long CSI, No response Limit) as between-subject factor. However, again very few participants had enough trials in the N-2 response-confusion error conditions so that we dropped this level and used a simplified 2 × 2 × 2 × 3 design.

### Results

#### Data trimming

Data trimming proceeded for all groups identically as described in the “General methods” section. In the No response limit group, we additionally removed slow responses (> 3000 ms), together with the subsequent two trials.

Nine participants were excluded from the analysis due to systematic fast guessing (three belonging to the High time-pressure group, two to the Long CSI group, and four to the No response limit group). Furthermore, data from 11 participants were removed due to excessive inaccuracy (five in High time-pressure group, five in Long CSI group, one in No response limit group). As anticipated above, very few participants had enough trials for statistical analysis in the N-2 response-confusion error condition (only 10 participants overall). As such, we decided to remove the N-2 response-confusion error condition and use a simplified 2 × 2 × 2 × 3 design. 35 participants still did not have a sufficient number of trials in at least one of the conditions (12 in High time-pressure group, 13 in the Long CSI group, 10 in No response limit group). The final sample was thus composed of 89 participants (28 in the High-time pressure group, 28 in the Long CSI group, 33 in No response limit group).

#### Frequency of different error types

Following data trimming, the average error rate was 12.6% (SD 6.2%, range 4.3–34.7%). Among errors, on average 25.8% (SD = 7.6%, range 8.4–45.9%) were classified as response-confusion errors, whereas the remaining 74.1% were classified as task-confusion errors (SD = 7.6%, range 54.1–91.5%).

#### Proportion of task-selection failures and task-execution failures

We assumed that task-confusion errors would more often be due to failure in task selection compared to response-confusion errors (which are mainly due to the participants activating the correct task, but selecting the wrong response within this task-set). To empirically test this assumption for Experiment 3, we again employed MPT modelling. A visual representation of the model is presented in Fig. 1. The empirical response categories were “correct”, “response-confusion error” and “task-confusion error”. The equations representing this model were:

3 $$ \begin{gathered} p\left( {{\text{correct}}} \right) \, = \, T*R \, + \, \left( {1 - T} \right)*R*0.5 \hfill \\ p\left( {\text{task-confusion error}} \right) \, = \, T*\left( {1 - R} \right)*0.5 \, + \, \left( {1 - T} \right)*R \hfill \\ p\left( {\text{response-confusion error}} \right) \, = \, T*\left( {1 - R} \right)*0.5 \, + \, \left( {1 - T} \right)*\left( {1 - R} \right) \, *0.5 \hfill \\ \end{gathered} $$

As described for Experiment 2, after estimating the model’s parameters using these equations, the parameter estimates were used to infer how many classified task- and response-confusion errors would be due to task-selection failures.

Given the model’s equations, this proportion were computed as:

4 $$ \begin{gathered} p{\text{(task selection failure | task confusion error}}) = \frac{{\left( {1 - T} \right)*R}}{{T*\left( {1 - R} \right)*0.5 + \left( {1 - T} \right)*R}} \hfill \\ p{\text{(task selection failure | response confusion error}}){ } = { }\frac{{\left( {1 - T} \right)*\left( {1 - R} \right)*0.5}}{{T*\left( {1 - R} \right)*0.5 + \left( {1 - T} \right)*\left( {1 - R} \right)*0.5}} \hfill \\ \end{gathered} $$

The estimated parameters were *R* = 0.932 (SD = 0.05, range 0.727–0.991), and *T* = 0.934 (SD = 0.04, range 0.837—0.988). Using the formulas presented in (4), the probability that a classified task-confusion error was due to task-selection failure was estimated to be 65.9% (SD = 14.2%), whereas the probability that an error classified as response-confusion error was actually due to task-selection failure was 6.6% (SD = 3.6%).

#### Analysis of N-2 repetition costs

**Fig. 4:**
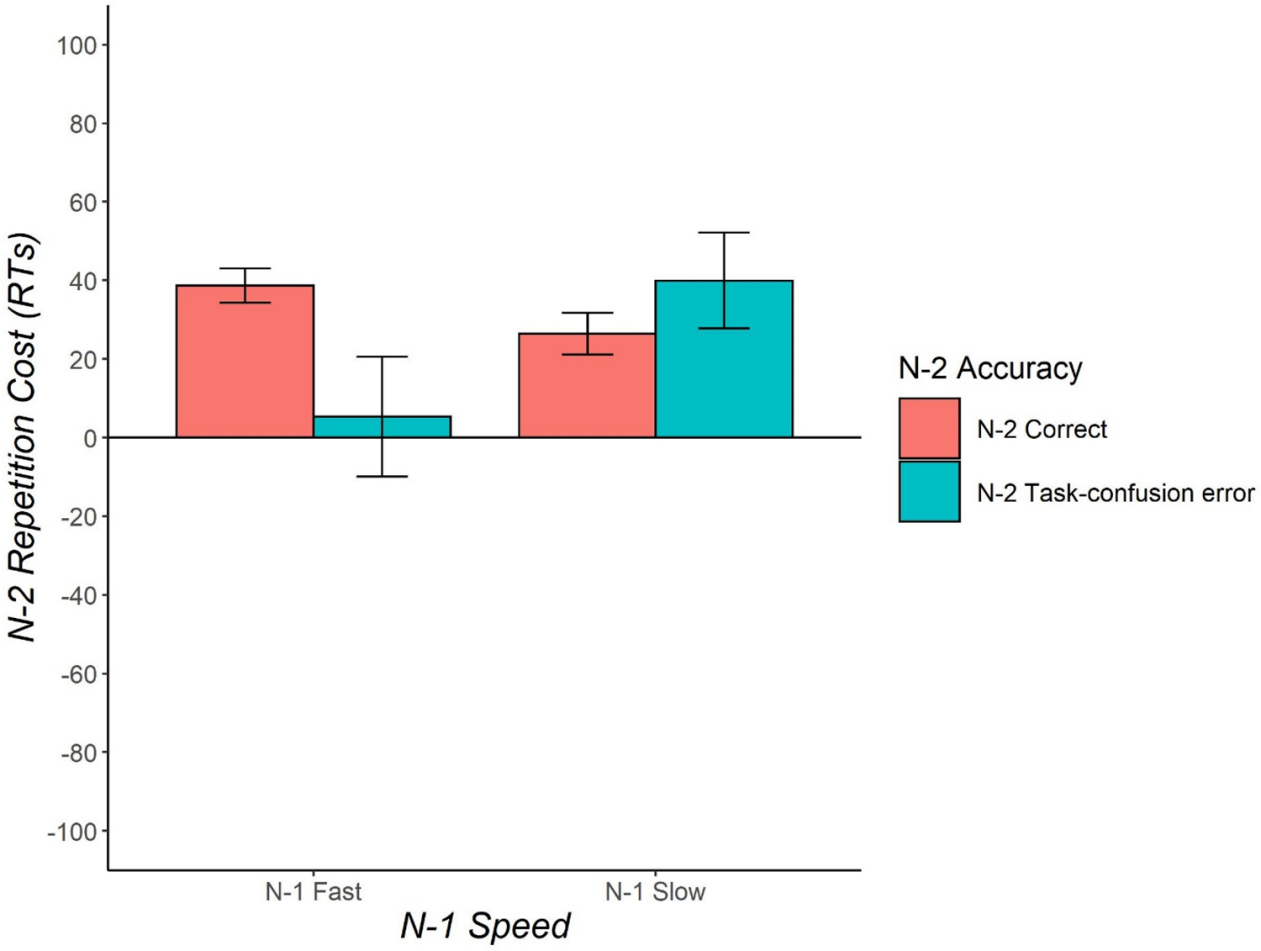
Experiment 3: Mean N-2 repetition costs (in ms) as a function of N-2 Accuracy (N-2 correct, N-2 task-confusion error), and N-1 Speed (N-1 slow, N-1 fast, as defined by median split on N-1 reaction times). Error bars indicate one standard error of the mean

The main effect of Group, *F*(2,86) = 19.59, *p* < 0.001, ${\eta }_{p}^{2}$ = 0.31, ${\eta }_{G}^{2}=.269$, revealed that RTs in the Long CSI group were shortest (466 ms), followed by the High time-pressure group (570 ms) and the No response limit group (698 ms). As in the other experiments, a main effect of Sequence indicated that N-2 repetition costs were found overall, *F*(1,86) = 25.53, *p* < 0.001, ${\eta }_{p}^{2}$ = 0.23, ${\eta }_{G}^{2}=.007$, while the main effect of N-1 speed confirmed again that trials following a slow response were slower than trials following a fast response, *F*(1,86) = 47.56, *p* < 0.001, ${\eta }_{p}^{2}$ = 0.36, ${\eta }_{G}^{2}=.029$. Most importantly, the critical 3-way interaction between Task Sequence, N-2 Accuracy, and N-1 Speed was significant, *F*(1,86) = 5.70, *p* = 0.019, ${\eta }_{p}^{2}=.06$, ${\eta }_{G}^{2}=0.001$, and is depicted in Fig. 4. This effect was not significantly modulated by Group, *F* < 1. When analysing the N-1 fast and N-1 slow conditions separately, we found a significant 2 × 2 interaction between N-2 Accuracy and Task Sequence in the fast N-1 trials, *F*(1,86) = 4.39, *p* = 0.039, ${\eta }_{p}^{2}=0.05$, ${\eta }_{G}^{2}=0.002$, which however was not strongly supported by the Bayes Factor, *BF*_*10*_ = 1.23. The interaction indicates that N-2 repetition costs were present following a correct response in N-2 (39 ms), *t*(88) = 8.91, *p* < 0.001, *d*_*z*_ = 0.95, BF_10_ > 100, but were absent following a task-confusion error (5 ms), *t* < 1, BF_10_ = 0.12. In N-1 slow trials, the same 2 × 2 interaction was instead absent, *F* < 1, BF_10_ = 0.26. Within this subset, N-2 repetition costs were again found following a correct response in N-2 (26 ms), *t*(88) = 4.97, *p* < 0.001, *d*_*z*_ = 0.53, BF_10_ > 100, as well as in the N-2 error condition (40 ms), *t*(88) = 3.28, *p* = 0.001, *d*_*z*_ = 0.35, BF_10_ = 16.24.

In addition to this predicted pattern of results, another significant 3-way interaction was found between Task Sequence, N-2 Accuracy, and Group, *F*(2,86) = 5.24, *p* = 0.007, ${\eta }_{p}^{2}=0.10$, ${\eta }_{G}^{2}=0.003$. When assessing the effect of task-confusion errors on N-2 repetition costs separately for each group, we observed that N-2 task-confusion errors tended to reduce N-2 repetition costs in the Long CSI group and No response limit group, but not in the High time-pressure group. For the details of this analysis, and an additional analysis not involving N-1 Speed, the reader is referred to the Appendix.

### Discussion

Generally speaking, the paradigm employed in Experiment 3 proved to be the most solid for disentangling errors due to failures in task selection vs. task execution (see below for a statistical comparison with Experiment 2). MPT modelling revealed that task-selection failures were indeed found to be present about 10 times more often in classified task-confusion errors than in classified response-confusion errors, which means that the empirical classification of error types in this paradigm is able to separate quite well the errors arising from different cognitive processing failures. It should also be noted, though, that classified task-confusion errors do not represent a “pure” measure of task-selection failures; rather, MPT modelling revealed that about 66% of classified task-confusion errors in Experiment 3 were “true” task-selection failures, whereas the remaining 34% were actually task-execution failures. Classified response-confusion errors, on the other hand, seem to provide a rather “pure” measure of task-execution failures: According to MPT modelling, 93.4% of classified response-confusion errors are “true” task-execution failures, whereas the remaining 6.6% were actually task-selection failures. We also compared the estimated proportions of task-selection failures between Experiments 2 and 3 (see below).

Regarding the N-2 repetition costs analysis, this paradigm still did not provide enough response-confusion errors for N-2 repetition costs to be analysed as a function of N-2 error type, thus forcing us to perform our planned comparisons on N-2 correct trials and N-2 task-confusion errors only. These analyses met our predictions: N-2 repetition costs were found to be absent (5 ms, BF_10_ = 0.12) following task-confusion errors in trial N-2, but as expected, this was the case only when the N-1 post-error trial was a fast one (note that this three-way interaction was significant in the frequentist ANOVA, but was not strongly supported by the more conservative Bayes factor analysis). Instead, when the N-1 post-error trial was slow, there was no evidence for any difference in N-2 repetition costs following N-2 correct or N-2 error trials (*F* < 1, *BF*_*10*_ = 0.26, for the interaction). N-2 repetition costs were observed in both conditions.

### Comparison of correctly classified task-selection failures among different methodologies

One aim of our study was to compare the different methodologies for disentangling errors due to task-selection failures and task-execution failures and to explore which of these methodologies provide well-suited empirical markers of these different error types. In the analyses of Experiments 2 and 3, we reported the mean estimated proportion of task-selection failures among the trials classified as task-confusion errors, and among the trials classified as response-confusion errors (- note that the paradigm applied in Experiment 1 does not allow for deriving such estimates). In the following, we compared these estimates directly across Experiments 2 and 3. We used permutation tests for the statistical comparison, as the distribution of our dependent variables deviated significantly from normality, and the sample in each experiment was not perfectly balanced, this way avoiding inflation of Type I error (Bradley, 1978). Performing independent sample *t* tests, however, showed a very similar result.

Regarding the category of task-confusion errors, the proportion of task-selection failures among task-confusion errors was significantly lower in Experiment 2 compared to Experiment 3, *p* = 0.007. Regarding the category of response-confusion errors, Experiment 2 contained a higher proportion of (wrongly classified) task-selection failures than Experiment 3, *p* < 0.001.

## General discussion

Compared to single-task studies, errors in multitasking paradigms can either be due to the incorrect selection of the task or to a failure in correctly carrying out the selected task. Distinguishing between these two error types is useful for elucidating the cognitive mechanisms behind error commission and for assessing the (differential) impact of these errors on subsequent performance. In the present study, we set out to use the three different methodologies employed so far in task-switching research for drawing this distinction. Applying MPT modelling, we tested how well each methodology provided an empirical marker of task-selection failures and task-execution failures. Importantly, being able to correctly detect these error types allowed us to assess their impact on N-2 repetition costs, which is an empirical marker of inhibitory control in task switching.

### A comparison of different methodologies

Generally speaking, the results of MPT modelling consistently revealed that it is possible to separate task-selection and task-execution failures into distinct empirical phenomena, which we name task-confusion error and response-confusion error, respectively. In particular, the models show very good results for unequivocally detecting errors due to task-execution failures: Among the labelled response-confusion errors, 86.5% (Experiment 2) to 93.4% (Experiment 3) are indeed due to task-execution failure. Consistent with previous literature, it is instead harder to tell whether a labelled task-confusion error is actually due to task-selection failure (Meiran & Daichman, 2005; Steinhauser & Hübner, 2006; Steinhauser et al., 2017). In these studies, the estimated number of correctly classified task-selection failures ranged from 19% (Meiran & Daichman, 2005) and 54.7% (Steinhauser & Steinhauser, 2019). As such, even though in our experiments there was still a considerable number of task-execution failures among classified task-confusion error, we believe our results to be very satisfying. In particular, the methodology employed in Experiment 3 proved advantageous in this respect, by showing that 65.9% of classified task-confusion errors were actually due to task-selection failure. In sum, we conclude that the methodology employed in Experiment 3 is superior in distinguishing the two error types.

As far as the use of univalent task-response mapping is concerned, we wish to stress that it is not possible with the data provided by Experiment 1 to draw a firm conclusion on its validity. In addition, to the best of our knowledge, this is one of only three studies using this methodology to distinguish task-selection and task-execution failures (Desmet et al., 2012; Meiran & Daichman, 2005), so that it is hard to establish a clear pattern of results. Nonetheless, we think it is interesting to notice that both in our study and in that of Meiran and Daichman (2005), the number of task-confusion errors was rather limited. This can either mean that univalent task-response mappings drastically diminish task-selection failures, or that the mapping of task-selection failures to wrong-effector responses is not warranted. While it is surely possible that competition between tasks is diminished by using univalent task-response mappings (Gade & Koch, 2007b; Kieffaber et al., 2013), it is worth noticing that N-2 repetition costs still occurred here and in at least one previous report (Costa & Friedrich, 2012), thus indicating that the previously relevant task-set must be inhibited to some degree for correct performance of the currently relevant task. We believe, together with Meiran and Daichman (2005), that using univalent task-response mapping may prove problematic as it confounds task preparation with effector preparation, thus resulting in strong effector pre-activation at the time of stimulus onset. This does not mean, however, that it is not possible at all to elicit task-confusion errors in this paradigm. In the study of Desmet et al. (2012), many task-confusion errors were obtained by increasing between-task conflict using invalid cues.

To conclude, the methodology employed in Experiment 3, using more response keys than levels of stimulus dimension, proved to be the most solid in disentangling errors due to task-selection and task-execution failures. This conclusion is suggested by the results of the permutation tests indicating that both task-execution and task-selection failures were correctly classified more often in Experiment 3 compared to Experiment 2. A similar conclusion can be derived from the existing literature showing that, on average, this methodology shows a higher prevalence of task-selection failures among classified task-confusion errors (~ 50%; see Steinhauser et al., 2017; Steinhauser & Steinhauser, 2019) than the congruency-based methodology used in Experiment 2 (~ 20%; Meiran & Daichman, 2005; Steinhauser & Hübner, 2006). We, therefore, recommend its deployment, when possible, for future studies in which disentangling task-selection and task-execution failures is needed.

### The impact of different error types on N-2 repetition costs

Having validated our assumptions concerning the mapping of task-selection and task-execution failures to distinct responses in our paradigms, we further proceeded with testing the impact of these error types on N-2 repetition costs. The general idea was to replicate the results obtained in a previous study from our group, showing that N-2 repetition costs are significantly reduced following an error in the N-2 trial, but only if RT in the N-1 trial was performed relatively fast (Moretti et al., 2021). As our previous study employed only incongruent stimuli, we aimed at extending these results, showing that this pattern would be present only for N-2 task-confusion errors, and not for N-2 response-confusion errors, as shown previously for reductions in task-switch costs following errors in trial N-1 (Desmet et al., 2012; Steinhauser & Hübner, 2006, 2008; Steinhauser et al., 2017).

Unfortunately, in most of our experiments, such direct comparison was not possible due to a lack of analysable task-confusion errors (in Experiment 1), or response-confusion errors (in Experiment 3). The only exception was Experiment 2, but even in this experiment, there was still a considerable number of subjects (~ 50% of the total) that had to be excluded due to a lack of response-confusion errors. We wish to underline here that part of the reason for the small number of analysable trials in these conditions was that many N-2 error trials were excluded during data trimming. Compared to other studies in the literature assessing the impact of errors on the task-switch costs (taking trial N-1 into account), our data trimming procedure was stricter, because we analysed the effects of N-2 error trials, and, therefore, had to exclude all N-1 error trials.

Nonetheless, we believe that the data reported in the present study produce valuable insights for task-switching and error-processing research. First, we find overall support for the idea that automatic task-set strengthening can be counteracted by a slowly building cognitive control process initiated upon error commission (Steinhauser & Hübner, 2008). In Experiment 3, we indeed found the expected 3-way interaction (at least in the frequentist ANOVA), showing that while “regular” N-2 repetition costs tend to decrease as a function of the time elapsed between trial N-2 and N (Gade & Koch, 2005; Koch et al., 2004; Scheil & Kleinsorge, 2014), N-2 repetition costs following task-confusion errors show the opposite pattern. Importantly, this was not shown in Experiment 1, where N-2 repetition costs after N-2 response-confusion error were of the same size in the N-1 Fast and N-1 Slow conditions.

While Experiments 1 and 3 thus provide good support for our predictions, the results of Experiment 2 go in the opposite direction. Here, we did not observe the predicted reduction of N-2 repetition costs after N-2 *task-confusion* errors in fast N-1 trials. Instead, we unexpectedly observed a reduction of N-2 repetition costs after N-2 *response-confusion* errors in fast N-1 trials (at least in the frequentist ANOVA, not supported by Bayes factors). There are only two differences between the previous (Moretti et al., 2021) and the present study. The first is that while in the previous study, we employed only incongruent stimuli, here both congruent and incongruent trials were used. It may be tempting to argue that the significant 3-way interaction found in the previous study was driven by task-execution failures in N-2, rather than being the result, as we assumed, of task-selection failures. After all, the MPT model in Experiment 2 shows that within this paradigm almost half of the classified task-confusion errors are still due to failures in task execution. However, we consider this possibility to be unlikely, as the same effect was replicated for task-confusion errors in Experiment 3 where the percentage of correctly detected task-selection failures among the empirical category of task-confusion errors was very high (i.e. 65.9%). The second difference between the present and the past study was that, to accommodate for an increased number of conditions, we doubled the length of the present experiment, compared to two out of three experiments in our previous study. This may have introduced some practice effects that were not observed in the previous study. An additional analysis exploring this possibility seems to point in this direction and can be found in the Online Appendix.

### Conclusion

To conclude, our study highlights the importance of carefully choosing a suitable method for mapping task-selection and task-execution failures to different observable events in task-switching. We argue that the methodology used in Experiment 3 is the most appropriate for this aim. In addition, we were able to provide further support for the existence of a cognitive control process initiated upon error commission, and aimed at counteracting the automatic strengthening of the erroneously selected task set.

## Supplementary Information

Below is the link to the electronic supplementary material.

Supplementary file1 (DOCX 915 kb)

## Data Availability

The raw data and analysis scripts can be downloaded from https://osf.io/zm5vf/.

## Footnotes

1 Marginal likelihoods for each model were calculated using a normal prior distribution on the effect size d with mean 0 and standard deviation σ_d_, where σ_d_ ~ Inverse χ^2^ (1, h^2^). The value of h^2^ must be set with caution as it determines the shape of the likelihood function on the effect size (i.e. it determines which effect sizes are expected to be likely a-priori according to the alternative hypothesis). We used h^2^ = 0.5, which is the default setting in the BayesFactor package for R (Rouder et al., 2017). Also, since the computation of marginal likelihoods is often computationally demanding, and Monte Carlo simulation is required for integration in relatively complex models such as ours (Rouder & Morey, 2012), we set the number of iterations to 1 million for each of the models.

2 As in all the experiments a large proportion of participants was excluded from analyses due to insufficient number of trials in some condition, we report some further analyses using a less stringent exclusion criterion in the Appendix.

## References

[CR1] Allport A, Styles EA, Hsieh S, Umiltà C, Moscovitch M (1994). Shifting intentional set: Exploring the dynamic control of tasks. Attention and performance XV: Conscious and nonconscious information processing.

[CR2] Allport A, Wylie G, Monsell S, Driver JS (2000). Task switching, stimulus-response bindings, and negative priming. Attention and performance XVIII: Control of cognitive processes.

[CR3] Anwyl-Irvine AL, Massonnié J, Flitton A, Kirkham N, Evershed JK (2020). Gorilla in our midst: An online behavioral experiment builder. Behavior Research Methods.

[CR4] Bakeman R (2005). Recommended effect size statistics for repeated measures designs. Behavior Research Methods.

[CR5] Batchelder WH, Riefer DM (1999). Theoretical and empirical review of multinomial process tree modelling. Psychonomic Bulletin & Review.

[CR6] Bradley JV (1978). Robustness?. British Journal of Mathematical and Statistical Psychology.

[CR7] Braem S, Bugg JM, Schmidt JR, Crump MJ, Weissman DH, Notebaert W, Egner T (2019). Measuring adaptive control in conflict tasks. Trends in Cognitive Sciences.

[CR8] Bugg JM, Crump MJ (2012). In support of a distinction between voluntary and stimulus-driven control: A review of the literature on proportion congruent effects. Frontiers in Psychology.

[CR9] Brysbaert M (2019). How many participants do we have to include in properly powered experiments? A tutorial of power analysis with reference tables. Journal of Cognition.

[CR10] Cochrane A, Simmering V, Green CS (2021). Modulation of compatibility effects in response to experience: Two tests of initial and sequential learning. Attention, Perception, & Psychophysics.

[CR11] Costa RE, Friedrich FJ (2012). Inhibition, interference, and conflict in task switching. Psychonomic Bulletin & Review.

[CR12] Desmet C, Fias W, Hartstra E, Brass M (2011). Errors and conflict at the task level and the response level. Journal of Neuroscience.

[CR13] Desmet C, Fias W, Brass M (2012). Preparing or executing the wrong task: The influence on switch effects. The Quarterly Journal of Experimental Psychology.

[CR14] Erdfelder E, Auer TS, Hilbig BE, Aßfalg A, Moshagen M, Nadarevic L (2009). Multinomial processing tree models: A review of the literature. Zeitschrift Für Psychologie/journal of Psychology.

[CR15] Gade M, Koch I (2005). Linking inhibition to activation in the control of task sequences. Psychonomic Bulletin & Review.

[CR16] Gade M, Koch I (2007). Cue–task associations in task switching. Quarterly Journal of Experimental Psychology.

[CR17] Gade M, Koch I (2007). The influence of overlapping response sets on task inhibition. Memory & Cognition.

[CR18] Gade M, Schuch S, Druey MD, Koch I, Grange JA, Houghton G (2014). Inhibitory control in task switching. Task Switching and Cognitive Control.

[CR19] Gade M, Souza AS, Druey MD, Oberauer K (2017). Analogous selection processes in declarative and procedural working memory: N-2 list-repetition and task-repetition costs. Memory & Cognition.

[CR20] Gluth S, Meiran N (2019). Leave-One-Trial-Out, LOTO, a general approach to link single-trial parameters of cognitive models to neural data. eLife.

[CR21] Grange JA, Kowalczyk AW, O'Loughlin R (2017). The effect of episodic retrieval on inhibition in task switching. Journal of Experimental Psychology: Human Perception and Performance.

[CR22] Grant DA, Berg E (1948). A behavioral analysis of degree of reinforcement and ease of shifting to new responses in a Weigl-type card-sorting problem. Journal of Experimental Psychology.

[CR23] Hu X, Batchelder WH (1994). The statistical analysis of general processing tree models with the EM algorithm. Psychometrika.

[CR24] Ikeda K, Hasegawa T (2012). Task confusion after switching revealed by reductions of error-related ERP components. Psychophysiology.

[CR25] Kieffaber PD, Kruschke JK, Cho RY, Walker PM, Hetrick WP (2013). Dissociating stimulus-set and response-set in the context of task-set switching. Journal of Experimental Psychology: Human Perception and Performance.

[CR26] Kiesel A, Steinhauser M, Wendt M, Falkenstein M, Jost K, Philipp AM, Koch I (2010). Control and interference in task switching: A review. Psychological Bulletin.

[CR27] Koch I, Gade M, Schuch S, Philipp AM (2010). The role of inhibition in task switching: A review. Psychonomic Bulletin & Review.

[CR28] Koch I, Gade M, Philipp AM (2004). Inhibition of response mode in task switching. Experimental Psychology.

[CR29] Koch I, Poljac E, Müller H, Kiesel A (2018). Cognitive structure, flexibility, and plasticity in human multitasking—An integrative review of dual-task and task-switching research. Psychological Bulletin.

[CR30] Kowalczyk AW, Grange JA (2019). The effect of episodic retrieval on inhibition in task switching: A diffusion model analysis. Psychological Research Psychologische Forschung.

[CR31] Lakens D (2013). Calculating and reporting effect sizes to facilitate cumulative science: A practical primer for t-tests and ANOVAs. Frontiers in Psychology.

[CR32] Mayr U, Keele SW (2000). Changing internal constraints on action: The role of backward inhibition. Journal of Experimental Psychology: General.

[CR33] Meiran N (1996). Reconfiguration of processing mode prior to task performance. Journal of Experimental Psychology: Learning, Memory, and Cognition.

[CR34] Meiran N (2000). Modeling cognitive control in task-switching. Psychological Research Psychologische Forschung.

[CR35] Meiran N, Daichman A (2005). Advance task preparation reduces task error rate in the cuing task-switching paradigm. Memory & Cognition.

[CR36] Meiran N, Gotler A, Perlman A (2001). Old age is associated with a pattern of relatively intact and relatively impaired task-set switching abilities. The Journals of Gerontology Series B: Psychological Sciences and Social Sciences.

[CR37] Meiran N, Kessler Y (2008). The task rule congruency effect in task switching reflects activated long-term memory. Journal of Experimental Psychology: Human Perception and Performance.

[CR38] Moretti L, Koch I, Steinhauser M, Schuch S (2021). Errors in task switching: Investigating error aftereffects in a task switching paradigm. Journal of Experimental Psychology: Learning, Memory, and Cognition.

[CR39] Norman, D. A., & Shallice, T. (1986). Attention to action. In: R. J. Davidson., G. E. Schwartz, & D. E. Shapiro (Eds.), *Consciousness and Self-Regulation* (pp. 1–18). Springer, Boston, MA.

[CR40] Olejnik S, Algina J (2003). Generalized eta and omega squared statistics: Measures of effect size for some common research designs. Psychological Methods.

[CR41] Peirce J, Gray JR, Simpson S, MacAskill M, Höchenberger R, Sogo H, Lindeløv JK (2019). PsychoPy2: Experiments in behavior made easy. Behavior Research Methods.

[CR42] Philipp AM, Jolicoeur P, Falkenstein M, Koch I (2007). Response selection and response execution in task switching: Evidence from a go-signal paradigm. Journal of Experimental Psychology: Learning, Memory, and Cognition.

[CR43] Pronk T, Wiers RW, Molenkamp B, Murre J (2020). Mental chronometry in the pocket? Timing accuracy of web applications on touchscreen and keyboard devices. Behavior Research Methods.

[CR44] Ridderinkhof KR (2002). Micro- and macro-adjustments of task set: Activation and suppression in conflict tasks. Psychological Research Psychologische Forschung.

[CR45] Ridderinkhof KR, van den Wildenberg WP, Wijnen J, Burle B, Posner MI (2004). Response inhibition in conflict tasks is revealed in delta plots. Cognitive neuroscience of attention.

[CR46] Riefer DM, Batchelder WH (1988). Multinomial modelling and the measurement of cognitive processes. Psychological Review.

[CR47] Rogers RD, Monsell S (1995). Costs of a predictable switch between simple cognitive tasks. Journal of Experimental Psychology: General.

[CR48] Rouder JN, Morey RD (2012). Default Bayes factors for model selection in regression. Multivariate Behavioral Research.

[CR49] Rouder JN, Morey RD, Verhagen J, Swagman AR, Wagenmakers EJ (2017). Bayesian analysis of factorial designs. Psychological Methods.

[CR50] Rubinstein JS, Meyer DE, Evans JE (2001). Executive control of cognitive processes in task switching. Journal of Experimental Psychology: Human Perception and Performance.

[CR51] Scheil J, Kleinsorge T (2014). N− 2 repetition costs depend on preparation in trials n− 1 and n− 2. Journal of Experimental Psychology: Learning, Memory, and Cognition.

[CR52] Schuch S, Dignath D, Steinhauser M, Janczyk M (2019). Monitoring and control in multitasking. Psychonomic Bulletin & Review.

[CR53] Schuch S, Koch I (2003). The role of response selection for inhibition of task sets in task shifting. Journal of Experimental Psychology: Human Perception and Performance.

[CR54] Schuch S, Koch I (2004). The costs of changing the representation of action: Response repetition and response-response compatibility in dual tasks. Journal of Experimental Psychology: Human Perception and Performance.

[CR55] Sexton NJ, Cooper RP (2017). Task inhibition, conflict, and the n-2 repetition cost: A combined computational and empirical approach. Cognitive Psychology.

[CR56] Sinai M, Goffaux P, Phillips NA (2007). Cue-versus response-locked processes in backward inhibition: Evidence from ERPs. Psychophysiology.

[CR57] Singmann H, Kellen D (2013). MPTinR: Analysis of multinomial processing tree models in R. Behavior Research Methods.

[CR58] Steinhauser M, Gade M (2015). Distractor onset but not preparation time affects the frequency of task confusions in task switching. Frontiers in Psychology.

[CR59] Steinhauser M, Hübner R (2006). Response-based strengthening in task shifting: Evidence from shift effects produced by errors. Journal of Experimental Psychology: Human Perception and Performance.

[CR60] Steinhauser M, Hübner R (2008). How task errors affect subsequent behavior: Evidence from distributional analyses of task-switching effects. Memory & Cognition.

[CR61] Steinhauser M, Maier ME, Ernst B (2017). Neural correlates of reconfiguration failure reveal the time course of task-set reconfiguration. Neuropsychologia.

[CR62] Steinhauser R, Steinhauser M (2019). Error-preceding brain activity links neural markers of task preparation to cognitive stability and flexibility. NeuroImage.

[CR63] Stuss DT, Shallice T, Alexander MP, Picton TW (1995). A multidisciplinary approach to anterior attentional functions. Annals of the New York Academy of Sciences.

[CR64] Stuss DT, Alexander MP (2007). Is there a dysexecutive syndrome?. Philosophical Transactions of the Royal Society B: Biological Sciences.

[CR65] Van Den Wildenberg WP, Wylie SA, Forstmann BU, Burle B, Hasbroucq T, Ridderinkhof KR (2010). To head or to heed? Beyond the surface of selective action inhibition: A review. Frontiers in Human Neuroscience.

[CR66] Vandierendonck A, Liefooghe B, Verbruggen F (2010). Task switching: Interplay of reconfiguration and interference control. Psychological Bulletin.

[CR67] Yamaguchi M, Proctor RW (2011). The Simon task with multi-component responses: Two loci of response–effect compatibility. Psychological Research Psychologische Forschung.

[CR68] Zheng X, Roelofs A, Farquhar J, Lemhöfer K (2018). Monitoring of language selection errors in switching: Not all about conflict. PLoS ONE.

