# Supplementary material for: Disentangling task-selection failures from task-execution failures in task switching: an assessment of different paradigms
Source: Psychol Res. 2022 Jul 14;87(3):929–50. doi: 10.1007/s00426-022-01708-5 (PMC10017612; doi:10.1007/s00426-022-01708-5)
Supplement: Supplementary file 1 — Supplementary file1 (DOCX 915 kb) [file 426_2022_1708_MOESM1_ESM.docx]

**Online Appendix**

**Moretti, Koch, Steinhauser, & Schuch (2021). Disentangling task-selection failures from task-execution failures in task switching.**

**Overview of Online Appendix**

Table A1: Table summarising all the N-2 repetition cost results reported in the main paper.

I. Analysis of N-2 repetition costs as a function of N-2 error, without considering the factor of N-1 speed

II. Analysis of practice effects on N-2 repetition costs: Analyzing first half of experiment only

III. N-2 repetition cost analyses with less stringent exclusion criteria

IV. Analysis of post-error behavioral adjustments

**Table 1A.** Summary of the results of the highest-order ANOVAs for each experiment, reported in the main text. Bayes factors in favour of the alternative hypothesis (BF_10_) are reported for Experiment 1 only as they were not computed for the highest-order ANOVA of Experiment 2 and 3.

|  | **Effect** | **F** | **p** | $\boldsymbol{\eta}_{\boldsymbol{p}}^{\boldsymbol{2}}$ | **BF_10_** |
| --- | --- | --- | --- | --- | --- |
| **Experiment 1** |  |  |  |  |  |
|  | Sequence | 6.72 | .016* | .22 | 5.75 |
|  | N-2 Accuracy | < 1 | .881 | .< .01 | 0.16 |
|  | N-1 Speed | 5.33 | .030* | .19 | 2.90 |
|  | Sequence x N-2 Accuracy | 3.18 | .088 | .12 | 0.64 |
|  | Sequence x N-1 Speed | < 1 | .718 | < .01 | 0.23 |
|  | N-2 Accuracy x N-1 Speed | 11.02 | .003** | .32 | 6.33 |
|  | Sequence x N-2 Accuracy x N-1 Speed | < 1 | .705 | < .01 | 0.31 |
| **Experiment 2** |  |  |  |  |  |
|  | Sequence | 22.99 | < .001 *** | .53 |  |
|  | N-2 Accuracy | 2.62 | .121 | .12 |  |
|  | N-1 Speed | 17.91 | < .001 *** | .47 |  |
|  | N-2 Congruency | 3.55 | .074 | .15 |  |
|  | Sequence x N-2 Accuracy | 1.42 | .248 | .07 |  |
|  | Sequence x N-1 Speed | < 1 | .356 | .04 |  |
|  | Sequence x N-2 Congruency | 1.03 | .322 | .05 |  |
|  | N-2 Accuracy x N-1 Speed | < 1 | .480 | .03 |  |
|  | N-2 Accuracy x N-2 Congruency | 2.21 | .153 | .10 |  |
|  | N-1 Speed x N-2 Congruency | < 1 | .345 | .04 |  |
|  | Sequence x N-2 Accuracy x N-1 Speed | 3.85 | .064 | .16 |  |
|  | Sequence x N-2 Accuracy x N-2 Congruency | < 1 | .584 | .02 |  |
|  | Sequence x N-1 Speed x N-2 Congruency | 4.78 | .041 * | .19 |  |
|  | N-2 Accuracy x N-1 Speed x N-2 Congruency | 2.00 | .173 | .09 |  |
|  | Sequence x N-2 Accuracy x N-1 Speed x N-2 Congruency | 1.42 | .247 | .07 |  |
| **Experiment 3** |  |  |  |  |  |
|  | Sequence | 25.54 | <.001*** | .23 |  |
|  | N-2 Accuracy | 3.56 | .063 | .04 |  |
|  | N-1 Speed | 47.56 | <.001*** | .36 |  |
|  | Group | 19.59 | <.001*** | .31 |  |
|  | Sequence x N-2 Accuracy | < 1 | .357 | .01 |  |
|  | Sequence x N-1 Speed | 1.45 | .233 | .02 |  |
|  | Sequence x Group | 3.04 | .053 | .07 |  |
|  | N-2 Accuracy x N-1 Speed | 11.40 | .001** | .12 |  |
|  | N-2 Accuracy x Group | < 1 | .787 | <.01 |  |
|  | N-1 Speed x Group | 2.07 | .133 | .05 |  |
|  | Sequence x N-2 Accuracy x N-1 Speed | 5.71 | .019* | .06 |  |
|  | Sequence x N-2 Accuracy x Group | 5.25 | .007** | .11 |  |
|  | Sequence x N-1 Speed x Group | 1.44 | .233 | <.01 |  |
|  | N-2 Accuracy x N-1 Speed x Group | < 1 | .888 | <.01 |  |
|  | Sequence x N-2 Accuracy x N-1 Speed x Group | < 1 | .940 | <.01 |  |

**I. Analysis of N-2 repetition costs as a function of N-2 error, without considering the factor of N-1 speed**

This series of analyses is identical to that reported in the main text, except that the N-1 Speed factor is not taken into account. Furthermore, error data are also analysed after being arcsine-transformed (Winer et al., 1971). The predictions are as in the main paper: we expect to find reduced N-2 repetition cost following a task-confusion error in trial N-2, but not after a response-confusion error in N-2.

The data trimming was identical to that for the analysis in the main paper. As such, in Experiment 1 the impact of N-2 response-confusion errors only was assessed, whereas in Experiment 3 the impact of N-2 task-confusion errors was under examination. In Experiment 2, the impact of both N-2 response-confusion errors and N-2 task-confusion errors was assessed, as both kind of errors could be observed (i.e. errors in congruent trials = response-confusion errors, errors in incongruent trials = task-confusion errors).

***Experiment 1*.** Descriptive statistics are reported in Figure A1. The ANOVA on RT data showed a significant main effect of Task Sequence, *F*(1,23) = 6.76, *p* = .016, $\eta_{p}^{2}$ = .23, $\eta_{G}^{2}=.014$, indicating the occurrence of N-2 repetition costs. The main effect of N-2 Accuracy was not significant, *F* < 1. The interaction between Task Sequence and N-2 Accuracy showed a trend toward significance, *F*(1,23) = 3.24, *p* = .085, $\eta_{p}^{2}$ = .12, $\eta_{G}^{2}=.004$. Post-hoc *t-*tests revealed significant N-2 repetition costs following a correct response in N-2 (44 msec), *t*(23) = 5.36, *p* < .001, *d_z_* = 1.09, but not following an N-2 response-confusion error (12 msec), *t* < 1.

The corresponding ANOVA on error data displayed again a main effect of Task Sequence, *F*(1,23) = 6.36, *p* = .019, $\eta_{p}^{2}$ = .22, $\eta_{G}^{2}=.030$, indicating that N-2 repetitions were more error prone than N-2 switches. The main effect of N-2 Accuracy approached significance indicating that N-2 errors were more likely to be followed by another error in trial N, *F*(1,23) = 4.07, *p* = .055, $\eta_{p}^{2}$ = .15, $\eta_{G}^{2}=.023$. The interaction between these variables was not significant, *F* < 1. N-2 repetition costs were found following an N-2 correct trial (1.3%), *t*(23) = 4.55, *p* < .001, *d_z_* = 0.93, and marginally so following an N-2 response-confusion error trial (2.8%) *t*(23) = 1.73, *p* = .097, *d_z =_* 0.35.


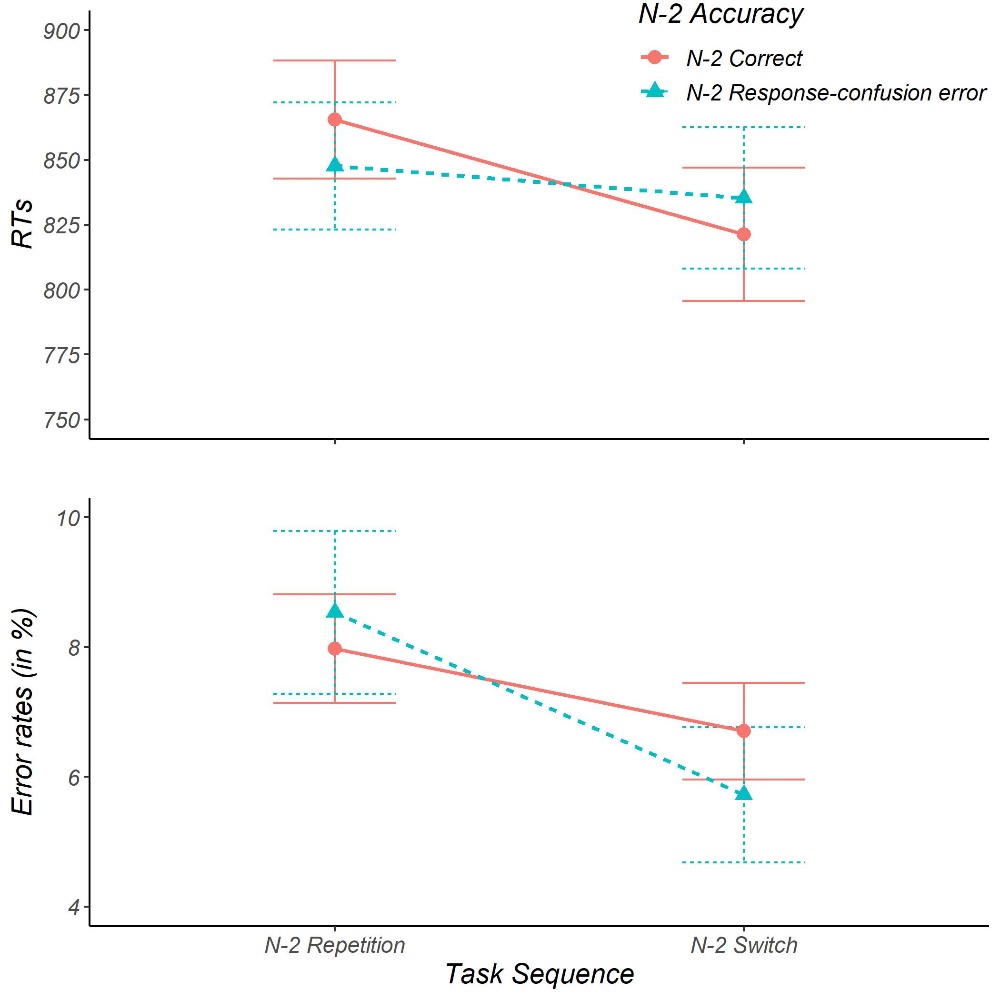


**Figure A1**. Experiment 1. Mean reaction times (upper panel, in ms) and mean error rate (lower panel, in %) as a function of N-2 Accuracy (N-2 correct, N-2 response-confusion error) and Task Sequence (N-2 Repetition, N-2 Switch). Error bars indicate standard error of the mean. Only response-confusion errors were included in this experiment.

***Experiment 2.*** Descriptive statistics for RTs and error rates are reported in Figure A2. In the ANOVA on RTs, only the main effect of Task Sequence was found to be significant, *F*(1,20) = 23.79, *p* < .001, $\eta_{p}^{2}$ = .54, $\eta_{G}^{2}=.017$, indicating the reliable occurrence of N-2 repetition cost. In addition, there was a trend for a main effect of N-2 Congruency, *F*(1,20) = 3.66, *p* = .070, $\eta_{p}^{2}$ = .15, $\eta_{G}^{2}=.004$, with slower responses for N-2 congruent trials.

The respective ANOVA on the square root arcsine transformed error rates revealed a strong main effect of N-2 Congruency, *F*(1,20) = 30.81, *p* < .001, $\eta_{p}^{2}$ = .60, $\eta_{G}^{2}=.062$, indicating increased error commission following congruent trials in N-2. Also, a main effect of N-2 Accuracy was present, *F*(1,20) = 8.72, *p* = .008, $\eta_{p}^{2}$ = .30, $\eta_{G}^{2}=.044$, with performance following an N-2 error being more error prone. No other effect reached significance.


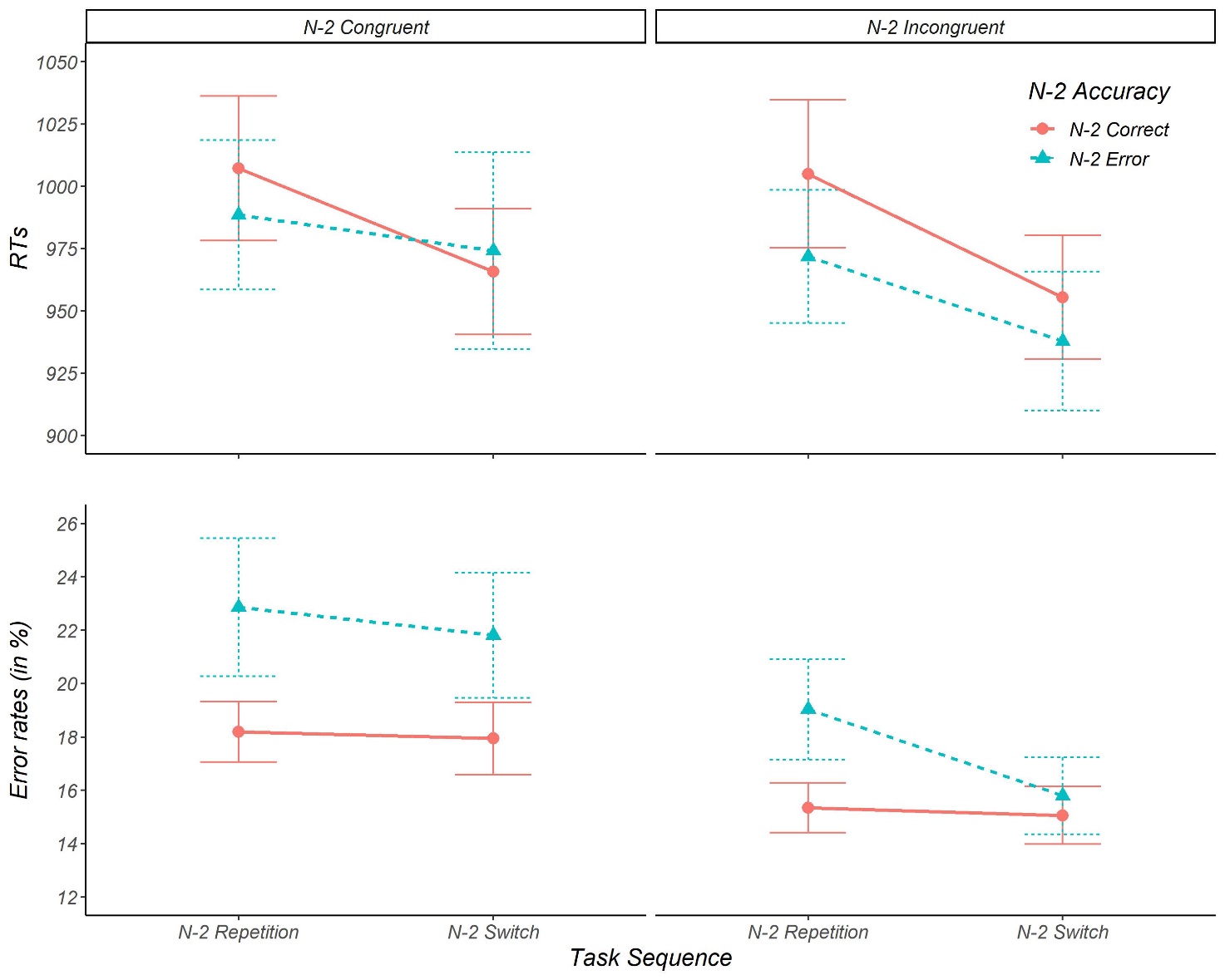


**Figure A2**. Experiment 2. Mean reaction times (in ms) as a function of N-2 Accuracy (N-2 correct, N-2 error), Task Sequence (N-2 repetition, N-2 switch) and N-2 Congruency (N-2 congruent, N-2 incongruent). Error bars indicate standard error of the mean. According to the experimental logic, errors in congruent trials correspond to response-confusion errors, while errors in incongruent trials correspond to task-confusion errors.

***Experiment 3****.* Descriptive statistics of both RT and error rates are reported in Figure A3. In RTs the within subjects ANOVA with N-2 Accuracy (N-2 correct, N-2 task-confusion error), Task Sequence (N-2 Repetition, N-2 Switch) and Group (High time pressure, Long CSI, No response limit) as factors revealed a solid main effect of Task Sequence *F*(1,86) = 26.19, *p* < .001, $\eta_{p}^{2}=.23$, $\eta_{G}^{2}=.008$. Also the main effect of Group was significant *F*(2,86) = 19.60, *p* < .001, $\eta_{p}^{2}=.31$, $\eta_{G}^{2}=.293$, with the High time pressure group being the fastest, followed by the Long CSI group and the No response limit group. Furthermore, N-2 Accuracy showed a trend toward significance *F*(1,86) = 3.80, *p* = .054, $\eta_{p}^{2}=.04$, $\eta_{G}^{2}=.004$, with N-2 error trials being marginally slower than N-2 correct. A Group x Task Sequence interaction was found *F*(2,86) = 3.13, *p* = .049, $\eta_{p}^{2}=.07$, $\eta_{G}^{2}=.002$, due to the fact that in the Long CSI group, N-2 repetition cost were significantly lower than in the other groups. Finally, the 3-way interaction involving all factors was also significant *F*(2,86) = 6.85, *p* = .007, $\eta_{p}^{2}=.11$, $\eta_{G}^{2}=.003$. In order to further investigate this interaction, separate 2 x 2 ANOVAs were carried out for each group. Only in the No response limit group the interaction between Task Sequence and N-2 Accuracy was significant *F*(1,32) = 5.71, *p* = .023, $\eta_{p}^{2}=.15$, $\eta_{G}^{2}=.005$. As expected, this interaction was due to N-2 repetition cost being well present following a correct response in N-2 (58 ms), *t*(32) = 8.33, *p* < .001, *d_z_* = 1.45, but not following an N-2 error (8 ms), *t* < 1.

In the error rates analysis, the main effect of Task Sequence *F*(1,86) = 13.55, *p* < .001, $\eta_{p}^{2}=.13$, $\eta_{G}^{2}=.013$, and Group *F*(2,86) = 2.89, *p* = .061, $\eta_{p}^{2}=.06$, $\eta_{G}^{2}=.041$, were in the same direction as in the RT analysis, as also the interaction between these two factors *F*(2,86) = 4.83, *p* = .010, $\eta_{p}^{2}=.10$, $\eta_{G}^{2}=.009$. Again, the 3-way interaction involving all factors was significant, *F*(2,86) = 3.12, *p* = .049, $\eta_{p}^{2}=.07$, $\eta_{G}^{2}=.009$. Contrary to the Long CSI and the No response limit groups, in the High time pressure group a significant interaction between Task Sequence and N-2 Accuracy was observed, *F*(1,27) = 8.94, *p* = .006, $\eta_{p}^{2}=.25$, $\eta_{G}^{2}=.017$. In this group, N-2 repetition costs were not found following a correct response in N-2 (0.5%), *t*(27) = 1.17, *p* = .250, *d_z_* = 0.22 but only following N-2 errors (5.2%), *t*(27) = 4.33, *p* < .001, *d_z_* = 0.82.

In summary, the analysis of N-2 repetition cost without taking into account N-1 Speed as factor never showed a significant interaction between N-2 Accuracy and Sequence. The only exception was constituted by the No response limit group in Experiment 3, which indeed displayed a significant reduction of N-2 repetition cost following N-2 errors in RTs. Furthermore, the same interaction, but with opposite direction was found in the error rates for the High time pressure group, indicating increased N-2 repetition cost following N-2 errors. As such, this data pattern appears to be quite mixed. We suggest that distributional analyses, such as that reported in the main text, are fundamental to clearly assess the impact of errors on N-2 repetition cost.


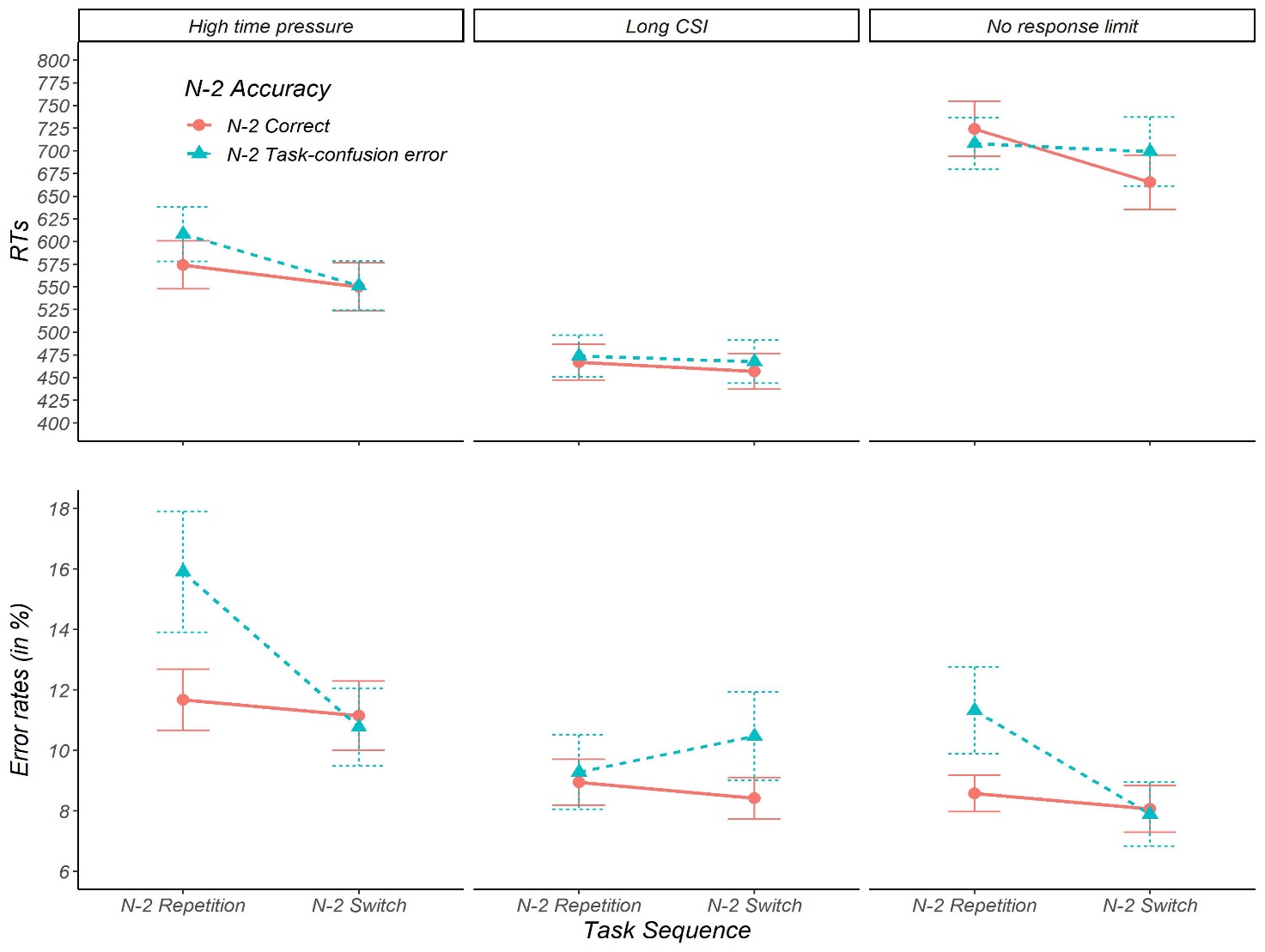


**Figure A3**. Experiment 3. Mean reaction times (in ms) as a function of N-2 Accuracy (N-2 correct, N-2 error), Task Sequence (N-2 repetition, N-2 switch) and Group (High time pressure, Long CSI, No response limit). Error bars indicate standard error of the mean. Only N-2 task-confusion errors were included in this experiment.

**II. Analysis of** **Practice effects on N-2 repetition costs: Analyzing first half of experiment only**

As outlined above, the most critical prediction in our study was to find reduced N-2 repetition cost following a task-confusion error in trial N-2, but only in the event in which the N-1 trial would belong to the fast half of the RT distribution. While this pattern of results was reliably found in three separate experiments in a previous study from our group (Moretti et al., 2021), results were mixed in the present study. This is particularly surprising when one considers that the

paradigm employed in Experiment 2 was almost identical to that employed in our previous study, the only differences being that Experiment 2 includes also congruent trials, and is twice as long as most of the experiments in our previous study.

In this exploratory analysis we therefore wish to investigate whether practice effects may have masked this well-replicated finding from our previous study. To this aim, we performed the same analysis including the N-1 Speed factor, but we analysed only the first half of each experiment. Figure A4 summarises the results for all experiments. For the sake of brevity, we will focus only on the highest order interaction for each ANOVA of the single experiments.


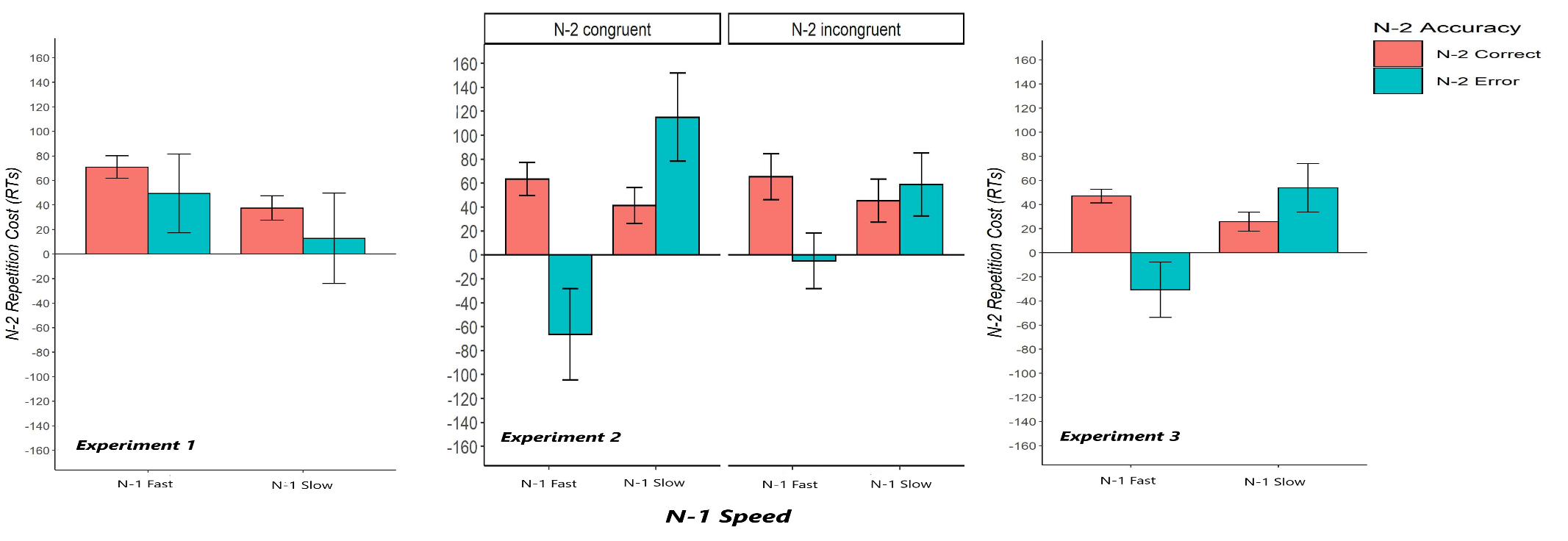


**Figure A4**. Analysis of first experimental halves only. N-2 repetition cost (RTs) as a function of N-2 Accuracy and N-1 Speed for Experiments 1 to 3. In Experiment 2, N-2 Congruency is considered as additional factor. In Experiment 1, only N-2 errors classified as response-confusion errors were included. In Experiment 2, the errors in N-2 congruent trials were classified as response-confusion errors, and the errors in N-2 incongruent trials as task-confusion errors. In Experiment 3, only N-2 errors classified as task-confusion errors were included in the analysis

***Experiment 1.*** In Experiment 1, where only N-2 response-confusion errors were analysed, no 3-way interaction was found, *F* < 1. This finding is in line with the prediction that N-2 repetition costs after N-2 response-confusion errors are not modulated by N-1 speed.

***Experiment 2.*** The 4-way interaction involving all factors (Task Sequence, N-1 Speed, N-2 Accuracy and N-2 Congruency) showed a trend toward significance, *F*(1,20) = 3.86, *p* = .063, $\eta_{p}^{2}=.16$, $\eta_{G}^{2}=.003$. Performing two separate ANOVAs, one for each level of the N-2 Congruency factor, we observed a significant 3-way interaction for the N-2 congruent trials, *F*(1,20) = 16.38, *p* < .001, $\eta_{p}^{2}=.17$, $\eta_{G}^{2}=.007$. As in the main analysis, the N-2 Accuracy and Task Sequence factors significantly interacted in the N-1 fast condition, *F*(1,20) = 10.01, *p* = .005, $\eta_{p}^{2}=.33$, $\eta_{G}^{2}=.046$, where N-2 repetition cost were found following N-2 correct trials (63 ms), *t*(20) = 4.59, *p* < .001, *d_z_* = 1.00, but even showed a tendency toward a facilitation following an N-2 error response (-66 ms), *t*(20) = -1.73, *p* = .098, *d_z_* = 0.38. In the N-1 slow condition there was still a tendency toward an interaction between N-2 Accuracy and Task Sequence, *F*(1,20) = 3.25, *p* = .086, $\eta_{p}^{2}=.14$, $\eta_{G}^{2}=.017$. However this time, the pattern was reversed: N-2 repetition cost were significant following a correct response in N-2 (41 ms), *t*(20) = 2.79, *p* = .011, *d_z_* = 0.61, but were even higher following an error (115 ms), *t*(20) = 3.12, *p* = .005, *d_z_* = 0.68.

Contrary to the main analysis, also in N-2 incongruent trials a marginally significant 3-way interaction was found, *F*(1,20) = 4.26, *p* = .052, $\eta_{p}^{2}=.32$, $\eta_{G}^{2}=.020$. The pattern of results is very similar to that found for the N-2 congruent trials, thus going in the expected direction. However, differences were less accentuated. In the N-1 fast condition we observed therefore again a reduction in N-2 repetition cost following an error in N-2, *F*(1,20) = 9.61, *p* = .006, $\eta_{p}^{2}=.32$, $\eta_{G}^{2}=.020$, going from 65 ms following a correct answer in N-2, *t*(20) = 3.40, *p* = .003, *d_z_* = 0.74, to virtually no difference (- 5 ms) *t*(20) < -1. In the N-1 slow condition instead, N-2 repetition cost were present both in N-2 correct (45 ms), *t*(20) = 2.51, *p* = .021, *d_z_* = 0.55, and N-2 error trials (58 ms), *t*(20) = 2.23, *p* = .037, *d_z_* = 0.48. Hence, when analyzing only the first half of the experiment, the predicted data pattern is observed, with reduced N-2 repetition cost following an N-2 incongruent error after N-1 fast trials, but no reduction of N-2 repetition cost following an N-2 incongruent error after N-1 slow trials. This predicted data pattern was not observed in the analysis of incongruent trials reported in the main paper, which included first and second half of the experiment, suggesting that increasing practice might alter this data pattern.

***Experiment 3.*** The critical 3-way interaction found in the main analysis was replicated when including only the first half of experimental trials, *F*(1,88) = 11.45, *p* = .001, $\eta_{p}^{2}=.12$, $\eta_{G}^{2}=.004$. In the N-1 fast condition, a significant interaction between Task Sequence and N-2 Accuracy was observed, *F*(1,88) = 10.65, *p* = .002, $\eta_{p}^{2}=.11$, $\eta_{G}^{2}=.007$, indicating again that N-2 repetition costs were present following N-2 correct responses (47 ms), *t*(88) = 8.34, *p* < .001, *d_z_* = 0.88, but were numerically even reversed following task-confusion N-2 errors (-30 ms), *t*(88) = -1.33, *p* = .186, *d_z_* = 0.14. On the contrary, in the N-1 slow condition, the same interaction did not reach significance *F*(1,88) = 1.89, *p* = .180, $\eta_{p}^{2}=.02$, $\eta_{G}^{2}=.001$. In this case, N-2 repetition costs were present both in the N-2 correct, (26 ms), *t*(88) = 3.26, *p* = .002, *d_z_* = 0.34 and N-2 task-confusion error condition (54 ms), *t*(88) = 2.68, *p* = .009, *d_z_* = 0.28. Hence, when analyzing only the first half of the experiment, the predicted data pattern was replicated in Experiment 3: N-2 repetition costs are reduced following an N-2 task-confusion error after N-1 fast trials, but not after N-1 slow trials.

**III.** **N-2 repetition cost analyses with less stringent exclusion criteria.**

In the main text we excluded from analyses of each experiment those participants who had less than 10 correct trials in each condition of the ANOVA design of interest, as was specified in the pre-registration protocols. Such criterion was chosen to ensure that we had relatively reliable estimates of mean performance for each participant in each condition, while keeping as many data as possible. Nonetheless, many participants had to be excluded: in Experiment 1, 20% of the total dropped out, in Experiment 2 roughly 50% and 25% in Experiment 3.

As a consequence, the statistical power of the ANOVAs reported in the main text may have been reduced (even though it is hard to assess the trade-off between large vs. reliable data on statistical power). For this reason, here we report the same analyses as those in the main text using a less stringent exclusion criteria: here, all participants with more than 5 trials in each ANOVA cell entered statistical analysis. Participants systematically using fast guesses or committing too many errors are instead excluded as before. Figure A5 summarises the results.

***Experiment 1.*** In Experiment 1 the new criteria led to the exclusion of 2 participants (instead of 6). As in the main text, the 3-way interaction between N-2 Accuracy, N-1 Speed and Sequence was not significant, *F* < 1, and support for the null was provided by bayesian analysis, *BF_10_* = 0.29.

***Experiment 2.*** In Experiment 2, 19 participants were excluded due to insufficient number of trials (instead of 31). As in the main text, the highest order 4-way interaction was not significant, *F* < 1. The only significant effects were those of Sequence *F*(1,32) = 25.79, *p* < .001, $\eta_{p}^{2}=.45$, $\eta_{G}^{2}=.014$, and N-1 Speed, *F*(1,32) = 18.11, *p* < .001, $\eta_{p}^{2}=.36$, $\eta_{G}^{2}=.024$. To further explore the impact of errors on N-2 repetition cost, follow-up ANOVAs were carried out on both N-2 Congruent and N-2 Incongruent trials. In neither of the two datasets did we find a significant 3-way interaction, *F*(1,32) = 1.98, *p* = .169, $\eta_{p}^{2}=.06$, $\eta_{G}^{2}=.001$, *BF_10_* = 0.4, and *F* < 1, *BF_10_* = 0.26, respectively.

***Experiment 3.*** In Experiment 3 only 2 participants did not have 5 trials in any of the conditions (instead of 28). The critical 3-way interaction between N-2 Accuracy, N-1 Speed and

Sequence was found to be significant, *F*(1,110) = 8.73, *p* = .004, $\eta_{p}^{2}=.07$, $\eta_{G}^{2}=.002.$ To this, we followed-up by assessing N-2 repetition cost as a function of accuracy in N-1 Fast and N-1 Slow trials separately. When the previous trial was fast a significant 2-way interaction was found, *F*(1,110) = 4.32, *p* = .040, $\eta_{p}^{2}=.04$, $\eta_{G}^{2}=.002$, even though not supported by bayesian analysis, *BF_10_* = 1.18. While N-2 repetition cost were robust following correct trials (38 ms), *t*(112) = 10.02, *p* < .001, *d_z_* = 0.94, *BF_10_* > 100, they were absent following an error, (5 ms), *t*(112) < 1, *p* = .765, *d_z_* = 0.03, *BF_10_* = .10. Also when the N-1 trial was slow we found a significant 2-way interaction, *F*(1,110) = 4.68, *p* = .032, $\eta_{p}^{2}=.04$, $\eta_{G}^{2}=.002$, again not supported by bayes factor, *BF_10_* = 1.41. Contrary to the pattern observed in N-1 fast trials, this interaction was driven by higher N-2 repetition cost following errors (56 ms), *t*(112) = 4.49, *p* < .001, *d_z_* = 0.42, *BF_10_* > 100, than following correct responses (27 ms), *t*(112) = 6.21, *p* < .001, *d_z_* = 0.58, *BF_10_* > 100.


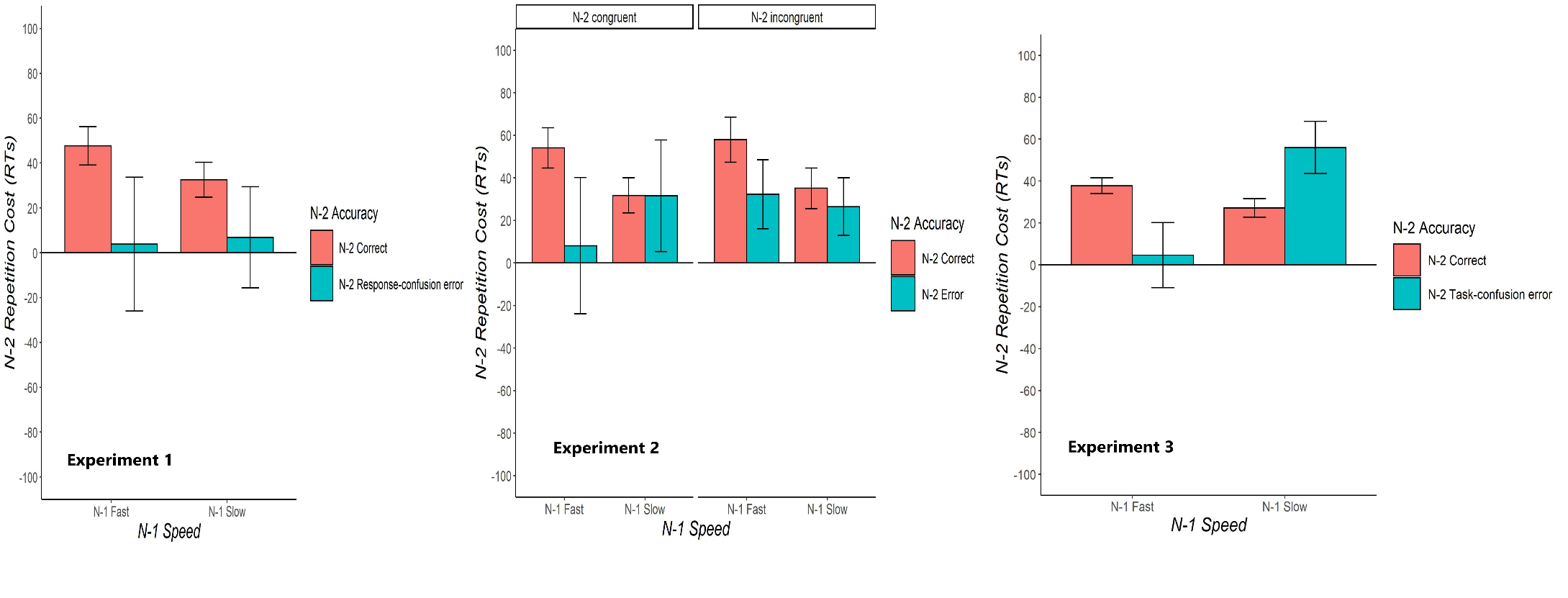


**Figure A5**. Analysis with less strict exclusion criteria. N-2 repetition cost (RTs) as a function of N-2 Accuracy and N-1 Speed for Experiments 1 to 3. In Experiment 2, N-2 Congruency is considered as additional factor. In Experiment 1, only N-2 errors classified as response-confusion errors were included. In Experiment 2, the errors in N-2 congruent trials were classified as response-confusion errors, and the errors in N-2 incongruent trials as task-confusion errors. In Experiment 3, only N-2 errors classified as task-confusion errors were included in the analysis.

**IV. Analyses of post-error behavioral adjustments**

While behavioral changes following error commission have been extensively studied in single-task context (Rabbitt, 1966), reports of post-error slowing (PES) or post-error changes in accuracy are much rarer in multitasking (Schuch et al., 2019). For this reason, we asked whether solid post-error behavioral adjustments would occur in a paradigm in which the task always switches from trial to trial.

For calculating PES, instead of comparing all post-error trials with all post-correct trials, we applied the procedure suggested by Duthil et al. (2012), comparing post-error trial RT with pre-error trial RT, thereby controlling for performance fluctuations over the course of the experiment that might otherwise compromise the measurement of PES. This meant that for each post-error trial, a “matching” pre-error trial had to be found, which needed to follow a correct response. Most of the times, this would be the trial right before the error [i.e. pre-error, error, post-error]. However, in some situations this was not possible. When there were two errors separated by just one correct trial for example, this would result in: error, post-error, error, post-error. As can be seen, the problem here is that the pre-error trial is “already” a post-error trial. In such situations, two matching pre-error trials were taken from the trials immediately before the first error. If this was not possible either because, for example an error was made at the beginning of a block, a random post-correct trial was selected from the same participant within the same block.

***Experiment 1.*** The paired *t-*test on RT showed a large PES (208 ms), *t*(23) = 8.80 , *p* <.001, *d_z_* = 1.79. The corresponding analysis on error data revealed a significant increase in error rates on trials following an error (4.0 %), *t*(23) = 3.63, *p* = .001, *d_z_* = 0.74.

***Experiment 2.*** PES was found in Experiment 2 as well (112 ms), *t*(20) = 6.79 , *p* <.001, *d_z_* = 1.48. Again, error rates significantly increased following an error (8.4%), *t*(20) = 3.56 , *p* = .002, *d_z_* = 0.77.

***Experiment 3.*** The pairted t-tests revealed a significant post-error slowing (137 ms), *t*(88) = 12.44, *p* < .001, *d_z_* = 1.31, as well as a significant decrease in accuracy following errors (6.4%), *t*(88) = 8.37, *p* < .001, *d_z_* = 0.89.

To summarize, across all three experiments, we consistently observed post-error slowing of 100-200 ms, with *d_z_* in the range of 1.3 to 1.8, together with a post-error decrease in accuracy of about 4% to 8%, *d_z_* about 0.7 to 0.9.

***References***

Dutilh, G., van Ravenzwaaij, D., Nieuwenhuis, S., van der Maas, H. L., Forstmann, B. U., & Wagenmakers, E. J. (2012). How to measure post-error slowing: a confound and a simple solution. *Journal of Mathematical Psychology*, *56*(3), 208-216.

Rabbitt, P. M. (1966). Errors and error correction in choice-response tasks. *Journal of Experimental Psychology*, *71*(2), 264-272.

Schuch, S., Dignath, D., Steinhauser, M., & Janczyk, M. (2019). Monitoring and control in multitasking. *Psychonomic Bulletin & Review*, *26*(1), 222-240.

Winer, B. J., Brown, D. R., Michels, K. M. (1971). *Statistical principles in experimental design.* New York: McGraw-Hill.
